# GeneHancer: genome-wide integration of enhancers and target genes in GeneCards

**DOI:** 10.1093/database/bax028

**Published:** 2017-04-17

**Authors:** Simon Fishilevich, Ron Nudel, Noa Rappaport, Rotem Hadar, Inbar Plaschkes, Tsippi Iny Stein, Naomi Rosen, Asher Kohn, Michal Twik, Marilyn Safran, Doron Lancet, Dana Cohen

**Affiliations:** 1 Department of Molecular Genetics, Weizmann Institute of Science, Rehovot 7610001, Israel; 2 LifeMap Sciences Inc, Marshfield, MA 02050, USA

## Abstract

A major challenge in understanding gene regulation is the unequivocal identification of enhancer elements and uncovering their connections to genes. We present GeneHancer, a novel database of human enhancers and their inferred target genes, in the framework of GeneCards. First, we integrated a total of 434 000 reported enhancers from four different genome-wide databases: the Encyclopedia of DNA Elements (ENCODE), the Ensembl regulatory build, the functional annotation of the mammalian genome (FANTOM) project and the VISTA Enhancer Browser. Employing an integration algorithm that aims to remove redundancy, GeneHancer portrays 285 000 integrated candidate enhancers (covering 12.4% of the genome), 94 000 of which are derived from more than one source, and each assigned an annotation-derived confidence score. GeneHancer subsequently links enhancers to genes, using: tissue co-expression correlation between genes and enhancer RNAs, as well as enhancer-targeted transcription factor genes; expression quantitative trait loci for variants within enhancers; and capture Hi-C, a promoter-specific genome conformation assay. The individual scores based on each of these four methods, along with gene–enhancer genomic distances, form the basis for GeneHancer’s combinatorial likelihood-based scores for enhancer–gene pairing. Finally, we define ‘elite’ enhancer–gene relations reflecting both a high-likelihood enhancer definition and a strong enhancer–gene association.

GeneHancer predictions are fully integrated in the widely used GeneCards Suite, whereby candidate enhancers and their annotations are displayed on every relevant GeneCard. This assists in the mapping of non-coding variants to enhancers, and via the linked genes, forms a basis for variant–phenotype interpretation of whole-genome sequences in health and disease.

**Database URL:** http://www.genecards.org/

## Introduction

Enhancers are *cis*-regulatory DNA sequences that are widely dispersed throughout genomes. Enhancers are distant-acting transcription factor (TF)-binding elements able to modulate target gene expression in a precise spatiotemporal specific manner (1, 2). There is considerable evidence that enhancer-based transcription regulation is involved in determining cell fate and tissue development (3, 4). The accepted model for enhancer-mediated activation of gene expression is that enhancers come into proximity with promoters by chromatin looping, thus recruiting the transcriptional machinery (1, 5–7). This mode of action is supported by chromosome conformation capture and related methods that detect direct interactions among remote chromatin regions (8). It is estimated that there are hundreds of thousands of enhancers in the human genome (9, 10), a count much larger than that of genes. Each enhancer binds several TFs, consistent with a combinatorial regulatory code (11), likely involving many-to-many relationships among enhancers and genes (12).

The accurate identification of enhancers is challenging (13), but recent progress has provided several relevant avenues to explore. The most direct approaches involve enhancer reporter assays, directed, for example, at non-coding DNA segments that show high interspecies sequence conservation (14, 15). An analogous experimental approach that has recently been applied on a high throughput scale is using massively parallel reporter assays (16, 17). Other methodologies that are likewise suitable for high-throughput genome-wide scrutiny are predictive in nature. These include the combined identification of several histone modification marks and DNase hypersensitive sites in different tissue types (18). Such an approach forms the basis of several genome-wide projects for enhancer identification and annotation (10, 19). Another relevant feature of active enhancers is that they undergo bidirectional transcription, forming enhancer RNA (eRNA) products (20, 21). The exact role of eRNAs remains elusive, but such measureable transcription signals have become effective tools for enhancer identification (22).

An even more challenging task is connecting enhancers with their target genes. Contrary to promoters that reside in the first 1–2 kilobases (kb) upstream from the transcription start site (TSS) of a gene, enhancers are often found dozens of kb away from the genes they influence, often across several intervening genes (23–25). The link between enhancers and genes is therefore much more difficult to determine. Early reports employed molecular genetics methodologies to determine an enhancer’s influence on a single gene (26). More recently, several predictive studies have been carried out to determine enhancer to gene links genome-wide, some utilizing a combination of several approaches (27, 28). Thus, eRNAs have been shown to be significantly co-expressed with the promoters that they regulate (22, 29). In parallel, chromosome conformation capture methodologies may be used to detect the proximity of an enhancer and its target gene, stemming from the physical looping of DNA (30). Finally, expression quantitative trait locus (eQTL) analyses may identify a link between the expression of a target gene and variations within an enhancer (31).

Enhancer-related diseases, referred to as enhanceropathies (32), may arise in two ways. The first is due to mutations in TFs that interact with the enhancer (33, 34), and the second is due to mutations within the enhancer regions themselves. One example of the latter are mutations in an enhancer connected with the sonic hedgehog gene (*SHH*), leading to developmental aberrations in preaxial polydactyly (35). Another is in a mutated enhancer for the β-globin gene cluster, causing thalassemia (36).

Clearly, there is a need for an integrated resource that unifies known human enhancers and their associated genes, making such data easily amenable to the biomedical research community (37). Therefore, in this study, we create a unified dataset of scored human enhancers based on a combination of several methods for enhancer identification, and based on data derived from numerous tissues. In parallel, we identify enhancer–gene association, again using the power of combining several complementary approaches. Other enhancer databases have been presented (38, 39), but they typically do not include the combination of multi-method enhancer integration, multi method gene–enhancer links, combinatorial scoring, removal of redundancy and incorporation into a readily available set of databases and genome interpretation tools, such as the GeneCards Suite.

## Materials and methods

### Enhancer mining and unification

Enhancers were mined from four sources:

1. Ensembl enhancers and promoter flanks from the version 82 regulatory build (19), based on datasets from ENCODE (10) and Roadmap Epigenomics (40).
2. FANTOM5 ‘permissive enhancers’ dataset from the Transcribed Enhancer Atlas (22).
3. Human enhancers from the VISTA Enhancer Browser accessed on 7 April 2016; This includes elements that show consistent cross-tissue reporter expression patterns in replicates (positive enhancers), as well as elements with weaker evidence (negative enhancers) (15). The latter are non-coding regions showing sequence or epigenome signatures that suggest functionality, but fail *in vivo* validation in mouse. Their inclusion has only a negligible effect on our analyses due to their small count (846). Also, these sequences may well be active at different embryonic time points than examined by VISTA, hence worthy of inclusion.
4. ENCODE proximal and distal enhancer regions (46 datasets) provided to ENCODE by the Zhiping Weng Lab, UMass (Supplementary Table S5) (10). Here, enhancer prediction relied on the identification of DNase hypersensitivity regions and histone H3K27 acetylation signals (http://zlab-annotations.umassmed.edu/enhancers/methods).

Data were processed differently for each source. All datasets were transferred to BED format and, apart from the Ensembl dataset (which was already in the latest genome build), subsequently converted to hg38 using CrossMap (41) using the UCSC Genome Browser (42) chain file. In some cases, enhancers were split into several sequences in the new genome build. In those cases, if the total length of the intervals between the split sequences was 2% or less of the total length of all sequences combined, then the sequences were treated as a single enhancer. Otherwise, the original enhancer, which was split in the new genome build, was not used in further analyses. For Ensembl, FANTOM5 and VISTA enhancers, we used data that underwent unification by the sources across all tissues and cell lines. For the ENCODE dataset, enhancer elements were only reported separately for 46 cell lines and tissue types, and such data often showed strong overlaps (e.g. Supplementary Figure S1). To attain uniformity of source utilization, we pre-processed the ENCODE data by performing across-tissue unification similar to that done by the other sources. The coverage for each nucleotide was computed with BEDtools version 2.25.0 (43). Every contiguous region with coverage of at least 2 was defined as an ENCODE enhancer, with redundancy level comparable to that of the other sources (Table 1).

**Table 1:** GeneHancer content

Enhancer source	Total number of elements	Mean length (bp)	SD length	Total genome coverage (bp)	Total genome coverage (%)	PMID
Ensembl	213 260	1080	1337	2.30E+08	7.18	25887522
FANTOM	42 979	289	163	1.24E+07	0.387	24670763
VISTA	1746	1784	1002	3.09E+06	0.0964	17130149
ENCODE^a^	176 154	1644	2071	2.90E+08	9.02	22955616
All sources combined	434 139	1233	1672	3.98E+08	12.4	This study
GeneHancer	284 834	1397	1934	3.98E+08	12.4	This study

Basic statistics of GeneHancer mined enhancer entities from four sources along with the integrated candidate enhancers. The ‘All sources’ row describes the combination of all mined enhancer elements before applying the GeneHancer unification algorithm.

a Data in the ENCODE row represent 1 742 514 original enhancer elements, which underwent pre-processing (see Materials and methods).

For the clustering procedure, enhancer elements from all of the above sources were used in order to define candidate enhancers. Overlaps between any number of enhancers from different sources were examined using BEDtools. Then, groups of overlapping enhancer elements were defined as candidate enhancers; a candidate enhancer’s start and end positions are based on the lowest start and highest end positions, within its group of enhancer elements. A similar procedure was utilized for comparison to a validation dataset from EnhancerAtlas (39). EnhancerAtlas data, ∼2.5 M enhancer elements reported separately in 105 tissues/cells, was downloaded from the EnhancerAtlas website, accessed on 12 January 2017. DENdb data, ∼3.5 M enhancer elements reported separately in 15 cell lines, was downloaded from the DENdb website, accessed on 15 December 2016.

For estimating the significance of the pairwise overlaps among enhancer sources, the numbers of overlapping and non-overlapping regions were computed for each source pair, taking into account the size of the human genome. We employed BEDtools using the fisher function. A two-sided *P*-value was calculated using Fisher's Exact Test Calculator for 2x2 Contingency Tables (http://research.microsoft.com/en-us/um/redmond/projects/mscompbio/fisherexacttest/). As the *P*-value was very low, the reported value is the upper bound of the true value. Additionally, we used the same methodology to test whether our clustered enhancers overlapped significantly with conserved regions from UCNE (a database of ultra-conserved non-coding elements) (44). All other analyses estimating significance of pairwise overlaps were performed similarly.

### Enhancer confidence score

Each enhancer in GeneHancer has a score representing a degree of confidence. The score is computed based on a combination of evidence annotations using the following components: (i) the proportion of supporting enhancer sources ($S_{sources}$ —count of sources divided by 4, the maximum number of sources); (ii) overlap with conserved regions from UCNE (44) ($S_{conserved}$, 1 for enhancers overlapping with a conserved region, 0 otherwise); and (iii) TFBSs score: for each enhancer, the count of unique TFs having a TFBS within the enhancer was calculated and normalized as follows: $S_{TFBS}=\frac{{log}_{10}(1+count)}{{log}_{10}(1+\max\left(count\right))}$. The TFBSs used for this calculation were mined from ENCODE ChIP-Seq datasets, as described in the ‘Transcription factor co-expression analysis’ paragraph in the Supplementary Methods. For candidate enhancers including enhancer elements derived from VISTA, those that showed consistent across-tissue reporter expression patterns (positive) were given an additional ‘VISTA boost’ ($S_{VISTA}-$a constant value of 0.25). Finally, the score of candidate enhancers including FANTOM eRNA elements included also an eRNA score, based on the maximum pooled expression of CAGE tag clusters for each FANTOM element (22). The eRNA score was calculated as log_10_ of the top eRNA value for each candidate enhancer, normalized by the genome-wide maximal eRNA score ($S_{FANTOM}=\frac{{log}_{10}(max(candidate\_fantom\_scores))}{4\cdot {log}_{10}(max(all\_fantom\_scores))}$). Thus, the overall GeneHancer confidence score (*S*_E_) was defined as:

(1) $$S_{E}=S_{sources}+S_{conserved}+S_{TFBS}+S_{VISTA}+S_{FANTOM}$$

### Gene–enhancer association and scoring

Gene–enhancer associations were generated based on five methods: eQTLs (45), eRNA co-expression (22), TF co-expression, capture Hi-C (CHi-C) (30) and gene target distance, all of which are described in the Supplementary Methods. Subsequently, a score *S*_GE_ was calculated for each gene–enhancer link, to estimate the strength of such connection. *S*_GE_ is defined as:

(2) $$S_{GE}=-{\text{Log}}_{10}\left(p_{g}\right)+S_{C}+c\cdot f$$

where *p_g_* is a combined *P*-value for eQTLs, eRNA co-expression and TF co-expression, computed by Fisher’s combined probability test via a χ^2^ test statistic (46). The second term (*S_C_*) represents the CHi-C score as provided by the source, constituting the logarithm of the ratio of observed to expected read counts (30). The third term is related to enhancer–gene distance, where *c* is a normalization score based on the average score from the first two terms across all gene–enhancer connections. To compute *f* we draw a gene–enhancer distance distribution (Supplementary Figure S8), and obtain *f* as the fraction of enhancers in the distance bin of the specific gene–enhancer pair. Gene–enhancer distances are computed between a gene’s TSS and the mid-point of an enhancer, and the distribution employed for the purpose of computing *f* excludes values from the CHi-C method, which lacks information in the crucial range of 0–20 kb.

Our method for computing *S*_GE_ is on the whole unbiased, and minimally involves arbitrary weighting factors. The three scores for eQTLs, eRNA co-expression and CHi-C are based on the reported summary statistics and the significance thresholds used in the original studies. For TF co-expression we computed *P*-values as shown in the Supplementary Methods (‘Transcription factor co-expression analysis’ paragraph). When possible, *P*-values were combined in a meta-analytic fashion, using the widely utilized Fisher’s combined probability test.

### Gene–enhancer network

Visualization of gene–enhancer networks (Figure 4) was produced using Cytoscape 3.4.0 (47). A *P*-value of the probability of finding an overlap between gene–gene pairs based on sharing (i) diseases or (ii) enhancers was calculated using a normal approximation to the hypergeometric probability, used in http://nemates.org/MA/progs/representation.stats.html. The number of possible pairs between the genes connected to either diseases or enhancers was used as ‘total genes’.

### Comparison to single-enhancer experimental studies

We constructed a set of 175 published cases of human functional regulatory regions confirmed by experiments (literature set) (Supplementary Table S4). The comparison set was built via three routes:

1. Use of the set of Mendelian regulatory mutations in Genomiser, obtained by careful manual curation of the scientific literature (48). The set contains 453 non-coding variants that underlie Mendelian disease, along with the relevant disease-causing genes, based on OMIM information. For the analysis we used 301 mutations, annotated by this source as residing within enhancers, promoters, and 5′-UTR, the latter including an appreciable number of suspected transcription regulatory elements (Supplementary Table S3). Redundancy was reduced by merging variants associated with the same gene and separated by ≤1 kb into a single case, leading to a total of 132 pairs of regulatory elements and genes employed in the analysis (Supplementary Table S3).
2. A set of 22 *invivo* validated heart enhancers and target genes from the cardiac enhancer catalogue (49).
3. Our own literature sampling, focusing on publications that experimentally identified a human enhancer and its gene target. This effort resulted with a set of 21 curated enhancer–gene pairs. When necessary, genome coordinates were converted to hg38 using CrossMap (41) and the UCSC Genome Browser (42) chain file. All records from the curated sets are described in Supplementary Table S4.

For the entire literature set, we examined: (i) whether a literature regulatory element overlaps with at least one of GeneHancer’s predicted enhancers and (ii) whether a literature target gene is identical to one of the GeneHancer targets for the overlapping enhancer. The statistical significance of the overall enhancer overlap was evaluated as described in the ‘Enhancer mining and unification’ paragraph of the Materials and methods section above.

### Data updates

GeneHancer, as part of the GeneCards Suite, is typically updated with each major version release three times a year (latest update November 2016, next update March 2017) and several minor versions in-between. In major releases, the entire GeneCards Suite knowledgebase is updated, including rebuilding the genes, diseases and enhancers sets and their relationships; minor versions incorporate localized improvements for particular suite members. We are currently exploring methods to include enhancers and their target genes in the minor versions where feasible. As the collection of datasets used to predict enhancers keeps growing [e.g. ChIP-seq data (50)], our future updates will benefit from improvements in our enhancer sources. In addition, our enhancer pipeline accommodates addition and integration of new data sources.

### Data availability

GeneHancer data are incorporated into the GeneCards database (51) and the GeneLoc human genome locator (52), making it freely available for educational and research purposes by non-profit institutions at http://www.genecards.org/ and https://genecards.weizmann.ac.il/geneloc/index.shtml, respectively. Data dumps are available online at the GeneLoc web site.

### Other technical details

Programs used: (i) BEDtools version 2.25.0 (43), (ii) CrossMap (41) and (iii) Cytoscape 3.4.0 (47). Programming languages: Perl, C, C# and Matlab (2016a).

## Results

### Enhancer mining and unification

**Figure 1:**
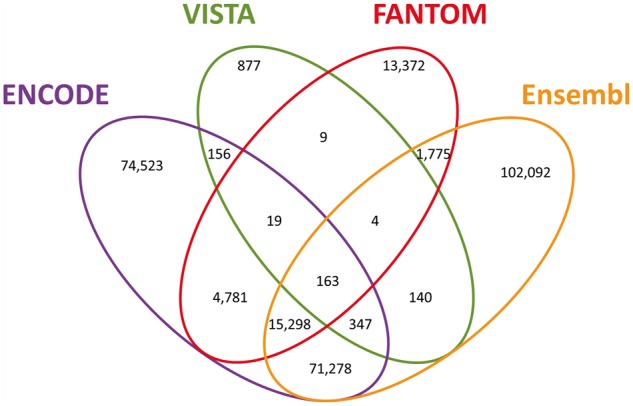
GeneHancer candidate enhancers. Venn diagram of the 284 834 candidate enhancers, split by the sources reporting each enhancer. Pairwise comparisons statistics (*P*, Fisher’s exact test *P*-value; OR, odds ratio; C, Clusters count): ENCODE–Ensembl (*P* = 8.1 × 10^−319^; OR = 19.6; *C* = 87 086), ENCODE–FANTOM (*P* = 1.9 × 10^−319^; OR = 17.1; *C* = 20 261), ENCODE–VISTA (*P* = 1.9 × 10^−140^; OR = 3.6; *C* = 685), Ensembl–FANTOM (*P* = 1.9 × 10^−319^; OR = 9.8; *C* = 17 240), Ensembl–VISTA (*P* = 5.8 × 10^−136^; OR = 3.5; *C* = 654), FANTOM–VISTA (*P* = 1.1 × 10^−51^; OR = 4.1; *C* = 195).

In order to generate a unified view of enhancers in the human genome, we mined data on enhancer identity and genomic locations from four sources (Table 1). For three of these datasets we mined lists of enhancer elements that already underwent tissue/cell line integration by the sources. For ENCODE, data were only available separately for each of 46 cell lines and tissue types. To attain uniformity of source utilization, we pre-processed the ENCODE data to create a non-redundant enhancer list (Materials and methods, Supplementary Figure S1). In total, the four sources yielded 434 139 unique tissue-integrated enhancer elements (Table 1). Examining the genomic coordinates of the across-source enhancer elements, we found that 56% (243 264) overlapped with at least one other enhancer derived from a different database, whereas 190 875 showed no overlap. To define a unified genome-wide enhancer set, we devised an algorithm that clusters enhancer elements based on their genomic locations. Following this procedure we obtained a list of 284 834 enhancer clusters (hereafter referred to as ‘candidate enhancers’), 33% of which were derived from more than one source (Figure 1, Supplementary Figures S1–S3). The overall degree of overlap across all sources was highly significant (Figure 1 legend).

The largest overlap of ∼87 000 enhancers is between ENCODE and Ensembl. This is to be expected, as these sources share common methodological factors, originating from common raw data, but differing in subsequent processing and analyses. Another portrayal of source overlap is that for each of the sources, the subset of elements in overlap with one or more of the others is between 47% and 62% (Supplementary Table S1), demonstrating good cross-source agreement even when the data have been derived via very different types of analyses. The highest number is observed for FANTOM (see also the ‘Comparison to VISTA’ paragraph below).

Each candidate enhancer is documented in the GeneHancer database, embedded within the GeneCards knowledgebase (51) and assigned a unique GeneHancer identifier. Furthermore, each enhancer is given a confidence score [Supplementary Figure S4 and Equation (1), Materials and methods], which is computed based on the number of supporting data sources, the number of unique TFBSs contained, and the overlap with ultra-conserved non-coding genomic elements. Additionally, we define ‘elite enhancers’ as those that have two or more evidence sources.

### Enhancer–gene associations

**Figure 2:**
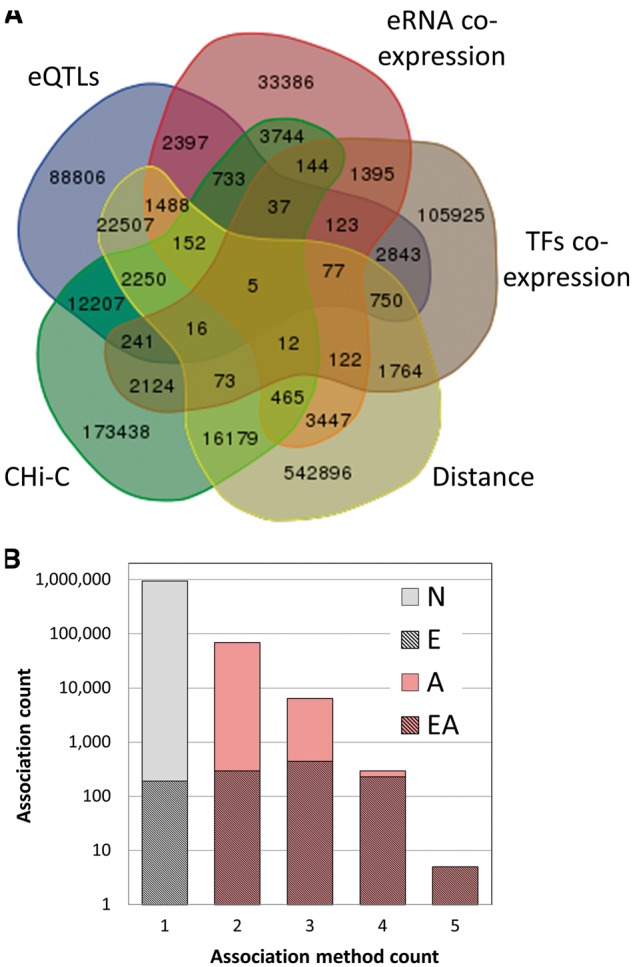
GeneHancer enhancer–gene associations. (**A**) Venn diagram of the 1 019 746 enhancer–gene pairs, grouped by the five distinct association methods. (**B**) Dependence of the count of gene–enhancer associations on the number of the relevant supporting methods. Gray, associations supported by one method only; pink, associations supported by multiple methods (elite associations); hatched, elite enhancers, with their proportion in each bin shown in a linear scale. ‘N’, no elite status; ‘E’, elite enhancer only (38% of total associations supported by one method); ‘A’, elite association only; ‘EA’, both elite enhancer and elite association (double elite). The proportions for double elite are 51%, 70%, 96%, 100% for method count 2, 3, 4 and 5, respectively.

**Figure 3:**
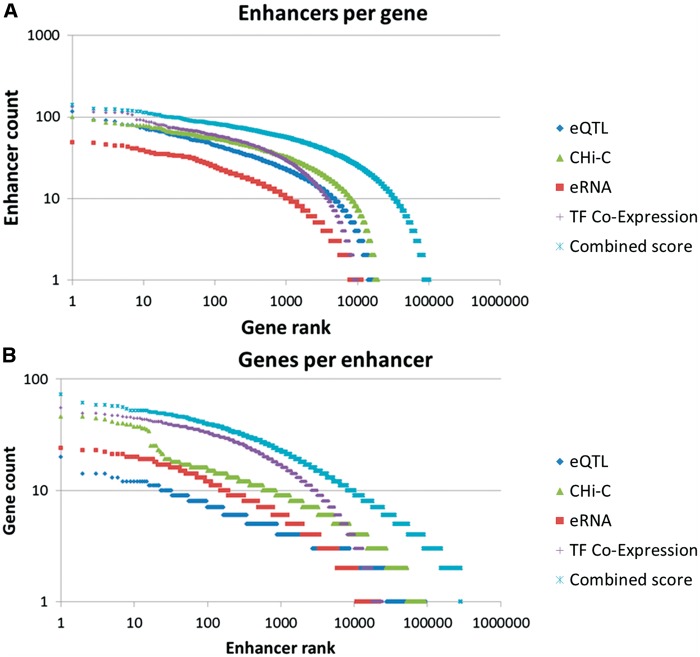
Gene–enhancer associations. Rank plots of (**A**) enhancer per gene counts and (**B**) gene per enhancer counts, using individual association methods and the combined method. The nearest neighbor method was not included in those charts since for most enhancers this approach promiscuously added its two flanking genes.

Subsequently, we explored evidence for relationships between candidate enhancers and genes. For this, we used five different methods that help infer gene–enhancer connections (Table 2, Figures 2 and 3), as detailed below.

**Table 2:** GeneHancer gene–enhancer associations content

Method	Number of connections	Connected genes	Connected enhancers	Genes per enhancer (average, SD)	Enhancer per gene (average, SD)	Multi-method connections	Multi-method proportion
eQTLs	134 632	18 028	93 482	1.44, 0.83	7.47, 8.26	45 826	0.34
CHi-C	211 820	19 461	92 621	2.29, 1.87	10.88, 10.46	38 382	0.18
eRNA	47 727	11 527	21 957	2.17, 1.88	4.14, 4.7	14 341	0.30
TF co-expression	115 651	10 040	24 569	4.71, 5.33	11.52, 13.13	9726	0.084
Nearest neighbor	592 203	97 400	284 821	2.08, 0.37	6.08, 6.61	49 307	0.083
All methods combined	1 019 746	101 337	284 821	3.58, 2.91	10.06, 11.8	75 295	0.074
Double elite associations	39 798	14 232	30 113	1.32, 0.77	2.80, 2.56	39 798	1

Basic statistics of associations based on five unique methods.

1. *eQTLs*. This entailed identifying single nucleotide polymorphisms (SNPs) within candidate enhancers, and then seeking correlations between enhancer SNP genotype and the expression of a potential target gene, as documented in the GTEx database (45). The validity of this approach was established by applying a similar eQTL analysis to a set of promoters that were uniquely affiliated to adjacent protein-coding genes (cognate genes). We found that in 33% of the cases, the promoter-mapped SNP had its best eQTL connection signal towards the expression of the cognate gene, providing a crude estimate for true positives in this analysis. Additionally, we found that enhancers had an eQTL density of 0.96 per kb whereas other genomic regions scored much lower (0.60 per kb). Additionally, although enhancers encompassed 0.184 of all eQTLs, they only contained 0.147 of non-eQTL SNPs (Fisher’s exact *P*=9.1 × 10^−318^, OR = 1.3), demonstrating that SNPs with targeted gene connections, are more likely to reside within enhancer than regular SNPs.
2. *C* *Hi-C*. We used a dataset of CHi-C (30), which maps regulatory interactions on a genomic scale for a set of 22 000 promoters as baits. We sought promoter-interacting fragments overlapping with our enhancer set. This allowed us to identify long-range interactions between 92 621 candidate enhancers and 19 461 promoter-proximate genes, resulting in 2.29±1.87 genes per enhancer.
3. *e* *RNA* *co-expression*. This was done with data from the FANTOM5 atlas of human enhancers (22). We recorded cases of co-expression between eRNAs transcribed from candidate enhancer regions and mRNA of potential target genes. We thus identified associations between 21 957 candidate enhancers and 11 527 genes, averaging 2.17±1.88 genes per candidate enhancer.
4. *TF* *co-expression*. This method focused on TFs that have binding sites determined by ChIP-seq analysis within a candidate enhancer (Supplementary Figures S5 and S6). We sought statistically significant correlations between the expression of such TFs and that of potential target genes for the candidate enhancers. This method supported associations between 24 569 candidate enhancers and 10 040 genes, averaging 4.71±5.33 genes per candidate enhancer. This approach was validated by two independent paths (see Supplementary Methods), each comparing pairwise cross-tissue expression correlations between a set of known TF-target gene pairs and random controls (Supplementary Figure S7). Both validations demonstrate a link between gene expression and the expression of TFs that regulate that gene, with statistical significance supported by the validation sets. This suggested that the co-expression metric we have defined can be used to assess enhancer–gene associations.
5. *Gene* *–* *enhancer distance (nearest neighbor links)*. Enhancer action is known to occur over considerable genomic distances (1), but the probability of regulatory events is thought to fall rather sharply with increasing gene–enhancer distances. This assertion is supported by an analysis of the distance behavior of all candidate gene–enhancer pairs obtained by the four methods described above (Supplementary Figure S8). To reflect such distance dependency, and following previously reported convention of focusing on immediate adjacency (15), we added a distance-based measure to the gene–enhancer pairing definitions. The immediately neighboring gene (not farther than 1 Mb) on each side of an enhancer is added, and the addition of these (typically) two genes per enhancer generates 542 896 new gene–enhancer connections.

In summary, the five enhancer–gene association methods helped define 1 019 746 connections amongst 284 821 candidate enhancers and 101 337 genes (genes per enhancer mean = 3.58 ± 2.91, enhancer per gene mean = 10.06 ± 11.8) (Table 2, Figures 2 and 3). To allow an assessment of the strength of the relation of a gene to a candidate enhancer, we developed a scoring formalism based upon measures derived from the five methods (Materials and methods, Supplementary Figure S9). We define ‘elite associations’ as cases in which a gene target for an enhancer is supported by two or more methods. Interestingly, we observe that the overlap between gene–enhancer association methods is quite modest. For each of the methods, the portion of associations having evidence also via one or more of the other methods is between 8% and 34% (Table 2). Overall, 7% of the gene–enhancer links are supported by multiple methods (elite associations). We further define a ‘double elite’ status as involving elite enhancers as well as elite associations (Figure 2B), reflecting a higher likelihood of prediction accuracy for both enhancer and target gene.

We also observe a relationship between the confidence scores of enhancer annotations and enhancer–gene associations, suggesting that the better supported enhancers show more consistent assignment to target genes (Supplementary Figure S10). Enhancers with higher annotation confidence, as reflected in the enhancer score we developed, not only possess more evidence for enhancer–gene association but also have more links annotated as elite gene–enhancer associations.

### Validation of enhancers and enhancer targets

#### Comparison to VISTA

When judging the degree of confidence in the ∼285 000 candidate enhancers in GeneHancer, one of the strongest criteria is whether an element receives support from at least two data sources. In all there are ∼94 000 such enhancers in our database, about 33% of the total number of enhancers. Our calculations show that the overlap is highly significant, attesting to the appreciable robustness of prediction by the individual sources. This also justifies a reliance on multisource enhancer assignment as one measure of the validity of such enhancer database entry.

VISTA is an experimentally verified enhancer source, not generated by predictive genome-wide techniques. Assuming that enhancers from VISTA have a high probability of being truly functional, we examined the correlation between the number of sources providing support for an enhancer (other than VISTA) and its inclusion in VISTA. The results indicate an increasing relative presence in VISTA when comparing enhancers with support from 1, 2 or 3 non-VISTA sources (Supplementary Figure S11 and Supplementary Table S2). Another indication for the significance of multi-source enhancer prediction arises from the observation that an increasing number of predicting sources leads to a higher fraction of cases with overlap to ultra-conserved non-coding genomic elements (44) (Supplementary Figure S12). The latter constitute 4351 genomic segments, 40% of which overlapped with our enhancers (Fisher’s exact *P* = 1.3 × 10^−320^, OR = 3.83).

We also asked how individual data sources differed in their overlap with VISTA, as a way of comparing the accuracy of their predictions. The results (Supplementary Table S1) show a relatively high proportion of FANTOM enhancers co-occurring with VISTA, when compared to the other two sources. This may suggest the strength of FANTOM as an enhancer predictive source, possibly related to its unique dependence on eRNA signals. The joint use of several sources combines this apparent advantage with the much larger coverage of Ensembl and ENCODE.

#### Comparison to single-enhancer experimental studies

In order to estimate the quality of the predicted enhancer set and enhancer–gene links in GeneHancer, we validated them against 175 published cases of human functional regulatory regions confirmed by experiments (literature set) (Supplementary Table S4). Our analyses encompassed three groups of such cases: (i) a set of 132 non-coding regulatory regions that harbor variants associated with Mendelian diseases, identified by manual curation of the literature (48) (Supplementary Table S3); (ii) a set of 22 *invivo* validated heart enhancers and target genes from the cardiac enhancer catalogue (49); and (iii) our own literature sampling, with 21 cases of experimentally identified enhancers and their gene targets.

There is significant agreement between the literature set and our predictions. As many as 69% of the published enhancers (121/175) overlap with the ∼285 000 enhancers in GeneHancer (Fisher’s exact *P* = 6.54 × 10^−56^, OR = 12.1). Furthermore, the scores of GeneHancer elements confirmed by the literature set are markedly higher than of those found in the entire GeneHancer dataset (Supplementary Figure S13), strengthening the validity of using our enhancer score to estimate candidate enhancer confidence. We further probed the agreement between the target genes of the literature set enhancers and those of their matched GeneHancer predictions. Rewardingly, 83% (100/121) of the literature target genes were identical to one of the target genes of our predicted enhancers. Finally, 56% of the matched enhancer–gene pairing in the overlap set were elite gene–enhancer associations, as compared to 7.4% expected at random. All of those findings are indicative of the validity of our approach to predict and estimate the confidence of enhancers and their gene targets.

#### Comparison to EnhancerAtlas

We examined two enhancer databases [DENdb (38) and EnhancerAtlas (39)], not yet included in our GeneHancer integration, for use in validation. The predicted enhancers in these two data sources were computed by us to have a similarly high genome coverage (43% and 52%, respectively). As there is a large difference in the number of tissues/cells reported (15 and 105, respectively), we opted to focus on EnhancerAtlas (Supplementary Figure S14). Rewardingly, we found that 88.1% of the GeneHancer elements were confirmed by EnhancerAtlas. Further, examining GeneHancer’s double-elite set of gene–enhancer pairs, we observed an appreciable overlap (51.1%) to target genes identified by EnhancerAtlas.

We then proceeded to estimate the potential enrichment of GeneHancer by a future inclusion of EnhancerAtlas in our unification pipeline. We used the enhancer element count proportion in the overlap set (∼1.9 M in EnhancerAtlas vs ∼0.25 M in GeneHancer) to obtain a crude estimate that the ∼0.55 M enhancer elements found in EnhancerAtlas but not in GeneHancer would add ∼0.072 M elements to the ∼0.28 M elements already in GeneHancer, a ∼25% increase. We note that enriching GeneHancer by a quarter will result in a 2-fold increase in genome coverage (Supplementary Figure S14), a disproportionate promiscuity boost, which would have to be regarded with caution. By definition, none of the added elements would be defined in GeneHancer as elite, because such elements would receive support only from one source.

### Gene–enhancer network

**Figure 4:**
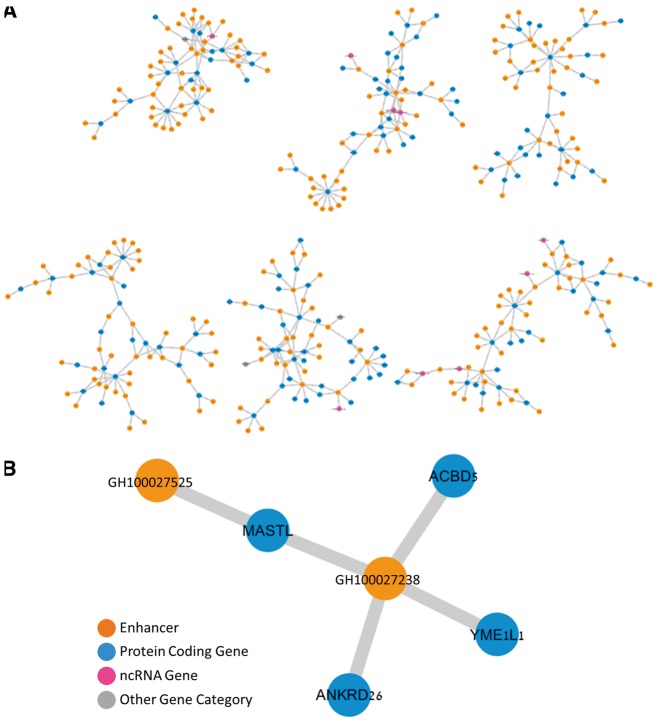
The gene–enhancer bipartite network in GeneHancer. (**A**) Representative six components of the ‘double elite’ (stringent) network, using elite enhancers and elite enhancer–gene pairs. (**B**) A single sub-network component with four genes and two enhancers, in which two of the genes, MASTL and ANKRD26, are linked to a mutual enhancer (GH100027238), and are also strongly associated with the Thrombocytopenia 2 disease.

The enhancer–gene connections described herein constitute a bipartite network with enhancers and genes comprising its two node types, and having ∼4000 connected components. This network has ∼10 enhancers per gene (Table 2), broadly consistent with the excess of enhancers over genes for the estimated 400 000 human enhancers (10) and ∼120 000 functional genes [∼20 000 protein coding genes and ∼100 000 non-coding RNA (ncRNA) genes (51)]. A state of multiple enhancers per gene also has ample literature support (53, 54). Interestingly, our database also depicts connections of more than one gene per enhancer, with a ∼3.6× ratio for the overall network (Table 2). Although such ratios show an opposite trend to the overall gene to enhancer ratio, they do have support by published evidence (55). Utilizing the ‘double elite’ definition, we obtain a more stringent network (Figure 4), with ∼30 000 enhancers, ∼14 000 genes and ∼8000 connected components. This network has ∼1.3 genes per enhancer and ∼2.8 enhancers per gene. The gene–enhancer links that do not appear in the elite network are made available to users with the caveat that they have a higher probability of not representing true regulatory events.

The stringent ‘double elite’ enhancer–gene network may also be used to derive indirect gene–gene relations. For example, the gene *MASTL* is connected, via a shared enhancer to three other genes (Figure 4B). One of these gene pairings (*MASTL* to *ANKRD26*) is corroborated by the fact that the same two genes are elite disease genes for the same disease (Thrombocytopenia 2) in MalaCards (56). This is one of 38 similar conjunction cases, much more than expected at random (normal approximation to the hypergeometric probability *P* < 6.0 × 10^−35^).

### Enhancers in GeneCards

GeneCards, the human gene compendium, is a gene-centric database with a web page for every human gene (51). Employing GeneCards’ integration philosophy, we built GeneHancer, aiming to judiciously unify, analyze and leverage the main sources of enhancers and enhancer–gene associations, and making them readily available to users via the GeneCards knowledgebase. GeneHancer is embedded within the relational database structure of GeneCards, thus facilitating portraying enhancer information for 101 337 genes, including 20 069 protein coding and 65 863 ncRNA genes (Supplementary Figure S15).

**Figure 5:**
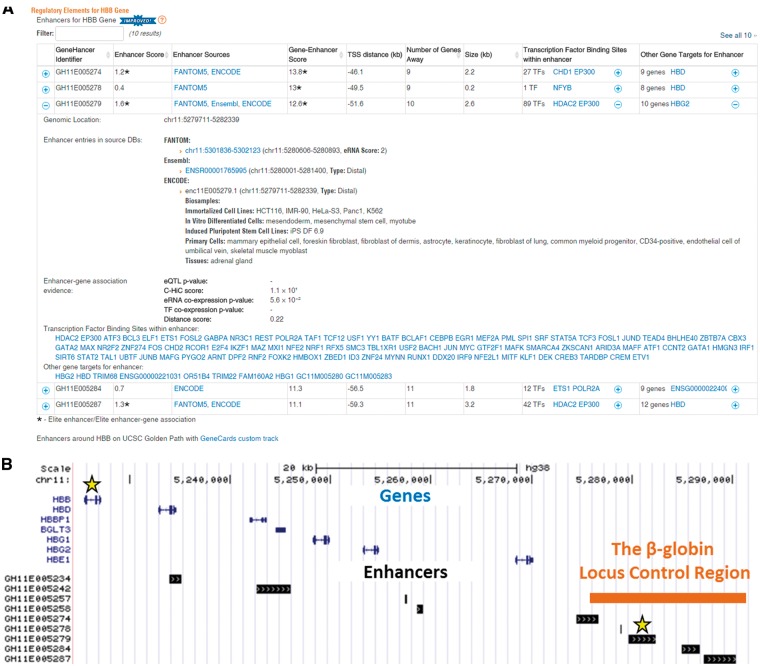
GeneHancer content in GeneCards. (**A**) An example of an enhancer table as portrayed in GeneCards for the HBB gene. Each row in this table describes a candidate enhancer associated with the HBB gene. For every enhancer, the following annotations are included: GHid (a unique and informative GeneHancer enhancer identifier, provided by the GeneLoc algorithm), confidence score (enhancers supported by two or more evidence sources are defined as elite enhancers and annotated accordingly with an asterisk), the sources with evidence for the enhancer, genomic size and a list of TFs having TFBSs within the enhancer. For every gene–enhancer association the following annotations are displayed: a general score for the gene–enhancer association (associations supported by two or more methods are defined to be elite and annotated accordingly with an asterisk), gene–enhancer distance (calculated between the enhancer midpoint and the gene TSS, positive for downstream and negative for upstream), number of genes having a TSS between the gene and the enhancer, and a list of other genes being associated with the enhancer. The expanded view, in this example for GH11E005279, shows also genomic location of the enhancer, and additional source-specific annotations such as identifiers, genomic locations, enhancer type (proximal/distal), a list of biological samples with evidence for the enhancer, eRNA expression strength (maximum pooled expression of eRNA CAGE tag clusters), tissue pattern and tissue pattern reproducibility. Additionally, the expanded view provides method-specific scores for the gene–enhancer association [*P*-values for eQTLs and co-expression, log(observed/expected) for CHi-C and distance-inferred probability score]. A link to a UCSC GeneCards custom track presenting all enhancers within 100 kb from the gene is located below the enhancers table. The screenshot was taken from GeneCards version 4.3 website. (**B**) GeneCards UCSC custom track view of the beta-globin locus. The enhancer expanded in the table, GH11E005279, is an elite enhancer with an elite association with HBB.

The GeneHancer content is displayed in a gene-centric fashion as a table in the ‘Genomics’ section of every GeneCard, providing users with a bird’s eye view of all enhancers of this gene along with their annotations (Figure 5A). This includes GeneHancer identifier, genomic location and length, enhancer confidence score, per-source enhancer information and the TFBSs contained within the enhancer genomic extent. For every enhancer we further show the strength of its association to the current gene (gene–enhancer score), gene–enhancer distance in both kb and in ‘genes away’ units and per-method enhancer–gene linking information. Also shown are the enhancer’s other associated genes. An option to download the enhancer table of a gene is under construction.

For visualization purposes, we generated a UCSC custom track linked from GeneCards (Figure 5B), which jointly displays all GeneHancer enhancers and genes in the selected genomic interval (defaulted to ±100 kb surrounding a selected gene). Of note, in the example shown, three of the five candidate enhancers overlapping with the beta globin locus control region (55) are elite enhancers (annotated with an asterisk), and they show a total of 10 elite associations (annotated with an asterisk) with the genes *HBB*, *HBD*, *HBG1*, *HBG2* and *HBE1*.

The GeneLoc tool (52), part of the GeneCards Suite, allows searching and browsing of GeneHancer data in a user friendly manner (https://genecards.weizmann.ac.il/geneloc/index.shtml, Supplementary Figure S16). The user can request a genomic interval by chromosomal coordinates, a megabase window around a gene, or an enhancer of interest. The results are in the form of a tabulated genomic map which includes enhancers and genes. One can further define map centers around genes or enhancers in the table. From the same GeneLoc table the user can click on a gene symbol to navigate to a specific GeneCard, which includes the aforementioned table of all enhancers linked to this gene. Similarly, clicking a GeneHancer identifier in the table activates a GeneCards search that shows all genes linked to that enhancer. Additional powerful searches within GeneCards allow querying by relevant TFs. An advanced search for tissues/cell lines relevant to an enhancer is being installed. A capacity to filter the GeneLoc table by such annotations, as well as by data sources, is under construction.

### Applications to whole-genome sequencing

Enhancer genomic aberrations have been reported to underlie human genetic diseases (57, 58), proposed to be included under the coined term enhanceropathies (32). One of the most notable examples is the deletion of the locus control region in thalassemia (59). A current challenge in the decipherment of the genetic underpinnings of human diseases is a capacity to tackle variations in regulatory elements and their related genes when performing medically oriented next generation sequencing. Addressing this challenge involves two different modes of analysis. The first is an ability to map variants to promoters and enhancers, which obviously necessitates whole-genome sequencing (WGS). The mapping program requires access to catalogues of promoters (e.g. Refs 19, 60, 61) as well as of enhancers, of which GeneHancer is an example. One of its advantages is that the enhancer coordinate compendium we have produced will be integrated into the WGS annotation and filtering functions of TGex, within the GeneCards Suite (62).

Having an informed variant annotation tool is necessary but not sufficient. Only in a few cases is there knowledgebase of information that directly links an enhancer to a disease/phenotype. Otherwise, the variant mapping step needs to be complemented by annotative information regarding a relationship between such an enhancer and a target gene, for which a phenotype relationship is already documented. In this realm, GeneHancer’s comprehensive integrated and scored set of gene–enhancer links is highly useful. It allows translating the finding of a WGS variant in a non-coding region into a variant-to-gene annotation, along with a confidence indication and a warning that this is not a direct inference. In fact, in the upcoming version of VarElect, the genome sequencing interpretation tool of the GeneCards Suite (62), we have decided to use only elite enhancer–gene relations, along with elite enhancer status, in line with the notion that basing scientific scrutiny only on one information source is risky. The inherent confidence score is supplemented by information in GeneHancer of accurate coordinates of TFBSs within each candidate enhancer element, whereby variants falling directly on a TFBS are much more likely to be pathogenic (63).

The involvement of regulatory elements in human disease may also result from functional aberrations in proteins, including TFs, that mediate enhancer function (32). An example of this is seen with *LEF1*, an enhancer binding TF associated with several malignant diseases such as leukemia (64). Thus, the TFBS content of enhancers (and promoters) is highly important for variant analyses of disease. In the more straightforward cases, direct knowledge is available linking the relevant TF gene to the disease or phenotype. In other cases, GeneHancer information, as portrayed in the GeneCards table (Figure 5A), provides vital links. This information is processed by VarElect in its indirect mode of action (62) as described below.

If a WGS (or exome sequencing) mutation is seen in a TF, VarElect is able to detect, through its search capacities, that both the TF (gene A), and a phenotype-associated gene (gene B) appear in the same enhancer element: Gene A—as indicated by a TFBS within such an enhancer, and Gene B as a target gene for the same enhancer. Thus, a TF sequence variant becomes linked to user-entered disease phenotype keywords in a process known as ‘guilt by association’ (62, 65). Importantly, this line of analysis symmetrically applies to cases in which a variant occurs in a candidate target gene, whereas the phenotype is linked to a TF gene. Finally, as the enhancer table includes indications for tissues in which such regulatory element is active, it becomes possible to use tissue or cell name strings to further pinpoint the phenotype search.

GeneHancer is useful for WGS analyses in several different ways. First, it goes far beyond regulatory elements for protein-coding genes, into the less charted realm of ncRNA regulatory elements (66). Thus, as many as 65% of ∼104 000 ncRNA gene entries in GeneCards have at least one associated candidate enhancer (Supplementary Figure S15). Furthermore, among the 14 232 genes in the stringent gene–enhancer network, 2820 are ncRNA genes. Second, WGS is especially effective in discovering copy number variations (CNVs), variants often associated with disease (67). As CNVs are much more likely to disrupt enhancer function than point mutations, enhancer information becomes especially relevant for a method tuned to efficiently discover CNVs.

## Discussion

### Multi-source unification

This article pertains to two major challenges: the first is collating data from different human enhancer databases to generate a unified compendium. The second is unifying information on gene–enhancer relationships from different data sources. Addressing both challenges has led to the generation of a usable knowledgebase on human candidate enhancers and their potential target genes. Because the methods utilized in the individual data sources are inherently noisy (68, 69), obtaining mutual support via unification has a significant potential for enhanced validity. This is beyond noise reduction obtained by data processing in some of the individual sources (19). We note, however that most of the enhancers in our database, particularly those supported only by one data source, are candidate enhancers that require future experimental validation.

The identification of individual enhancers typically constitutes the combinatorial application of several high-throughput genome-wide prediction methods. Widely used approaches involve chromatin signature profiling, including DNase hypersensitivity and histone modification signals, as well as sequence conservation and TFBS patterns. Our compendium handles data based on chromatin signatures and TFBSs from ENCODE and the Ensembl regulatory build. In addition, we include eRNA information from FANTOM and sequence conservation and *invivo* experimental validation data from VISTA. Bringing all of these points of evidence together is an advantage of GeneHancer.

Preliminary estimates suggested a set of ∼400 000 regions with enhancer-like features in the human genome (10). Our unification of four sources of data resulted in a total of ∼285 000 candidate enhancers, approaching three-fourths of the original estimated count. These enhancers encompass 12.4% of the length of the entire human genome, eight times higher than the coverage by coding exons. Thus, GeneHancer might constitute the largest compendium of non-redundant human candidate enhancers documented to date.

Other databases provide enhancer views based on multiple data sources or methodologies. One is DENdb (38), constructed based on several enhancer prediction techniques, and portraying a large number (∼3.5 million) of enhancer entries, suggesting considerable redundancy. This database also shows gene–enhancer links based on two approaches, chromatin interaction information and gene–enhancer distance. A second is EnhancerAtlas (39), which shows a similarly redundant collection of 2.5 million elements and provides analytic tools for determining gene–enhancer links. GeneHancer complements these databases via its major integration effort for both enhancers and enhancer–gene links, and through the advantages of portrayal in the GeneCards platform. The integration in GeneHancer stems from focusing on enhancers as genomic elements, as opposed to tissue-specific enhancer activities. As a result, the GeneHancer set of enhancers has only <300 000 elements, each annotated with tissue relationships.

### Promoters and enhancers

The GeneHancer database focuses on enhancers, and does not include declared promoter elements. This decision is guided by the notion that promoters are easier to identify by TSS proximity, and by the fact that numerous promoter databases already exist (10, 19, 60, 61). A confounding factor is that Ensembl and ENCODE invoke two enhancer types, distal and proximal, the latter also identified as ‘promoter flanks’ and representing potential promoters (19, 70). GeneHancer elements encompass both proximal and distal enhancers. This apparent ambiguity reflects a growing sentiment that enhancers and promoters are interrelated, sharing central molecular attributes, such as DNaseI hypersensitivity, histone modifications and transcriptional activity, and thus not easily distinguishable (71, 72). Such observations lend support to including ambiguous elements in an enhancer compendium even if they eventually turn out to be unambiguous promoters. Such broad inclusion principle goes hand-in-hand with the thought that our database entries are aimed to serve as pointers for further research and functional characterization.

In view of the enhancer–promoter interrelations, we have directly assessed the overlap between our candidate enhancers and promoters from an independent source, the Eukaryotic Promoter Database (EPD) (60), as well as from Ensembl. Interestingly, very high overlaps were found, whereby 82% of the EPD promoters and 98% of the Ensembl promoters overlapped with GeneHancer elements.

Using a more promiscuous promoter definition, namely the first upstream 1 kb from the TSS of every protein-coding or ncRNA gene, 38% of such presumed proximal regulators overlapped with GeneHancer elements, over two-thirds of which were not included in either EPD or Ensembl. These findings highlight the challenge in differentiating between promoters and enhancers, especially when heavily relying on histone marks, as exemplified by ENCODE. This overlap is also in line with reported difficulties in differentiation between enhancers and promoters via chromatin profiling assays (73).

### Enhancer–gene links

The identification of target genes for an enhancer is a challenging task. One of the reasons is that, in contrast to promoters, enhancers act across much larger distances, often spanning intervals with numerous potential gene candidates. The unified candidate enhancer compendium generated here forms a solid foundation for seeking gene–enhancer interactions. This was done by several different methods, and again, the principle of unifying several discovery avenues has been employed, as also described elsewhere (74). The methods included more direct inferences such as CHi-C or gene–enhancer distance, as well as indirect/indicative approaches such as genetic inference and co-expression analyses. It is therefore advisable to maximally rely on their combination, and to perform quality assurance steps, so as to increase reliability and reduce noise. This is reflected in the scoring system and elite status in GeneHancer. We note that at present a large majority (93%) of the gene–enhancer link inferences presented here are based on a single method. Such low overlap between methods questions the accuracy and reliability of single-method inferences. For this reason we have defined the double elite status as a default basis for our WGS analysis and interpretation tools VarElect and TGex.

One of the indirect methods utilized is eQTLs, based on genetic association (75, 76). Notably, an eQTL signal between a variant within an enhancer and a gene whose expression is modified does not guarantee that the gene is targeted by the enhancer. To address this limitation, we performed two analyses which provided an estimate for the robustness of the methodology. In the first analysis we used promoters as controls, showing that in as much as 33% of the cases the eQTL connected the regulatory element to its known cognate gene, an appreciable rate of accuracy. In a second analysis, we found that eQTLs were enriched in enhancers, both when comparisons were made with non-enhancer regions, and also with non-eQTL SNPs in enhancers.

Similar robustness analyses were conducted for the co-expression method, in which a correlation between the expression of enhancer-interacting TF and the candidate enhancer target gene was sought (74). The rationale of this method is that a TF that takes part in the enhancer complex must be up- or down-regulated in concordance with the target gene. Such analysis would be less accurate in cases where a TF participates in multiple transcription complexes, including involvement in house-keeping processes, or when combinatorial regulation happens, that involves several TFs. The robustness analyses performed included the examination of co-expression between TFs and candidate target genes, both in cases of known promoter-target gene pairs and for text-mined TF-target gene pairs (77), yielding supportive results.

Finally, the last two gene–enhancer pairing methods utilized (eRNA co-expression and CHi-C) were subjected to quality assurance elsewhere (22, 30). All of the above analyses provided further support for the gene–enhancer pairing methodologies employed in this article.

Among the plethora of available methodologies for determining genome-wide distant chromatin interactions, we opted for utilizing CHi-C. This method is highly suitable for linking enhancers to genes, as it allows focusing on gene promoters as baits, and exploring links to other DNA regions, typically restricted to distances up to 1 Mb. We note that the recorded physical links may not always represent gene–enhancer interactions. Yet, there are ample reports for the successful use of this and related chromosome conformation capture methods for such purposes (78, 79). To increase specificity, we opted to only use ≤10 kb resolution data from a specific source (30), which also provided false discovery rate cutoffs criteria. As average enhancer length in our data is 1.4 kb, and average inter-enhancer distance is ∼10 kb, the aforementioned length cutoff decreases the probability of mapping irrelevant enhancers to a promoter. The data used stem from only two cell types, so arguably could provide a very partial picture. However, a detailed analysis of the overlaps among the five methods for gene–enhancer links indicates that CHi-C is by no means an outlier in the degree of overlap (Figure 2, Table 2).

This article describes the use of several methodologies that provide a great deal of evidence on enhancer–gene links beyond the traditional genomic proximity. Still, we opted to judiciously use also the genomic distance (nearest neighbor) criterion, adding two immediately adjacent genes for every enhancer. We note that even prior to introducing the distance linking criterion, as many as 10% of all enhancer–gene connections were between immediately neighboring gene and enhancer, suggesting validity for the distance criterion. However, as a result of adding links to neighboring genes, the percentile rose to 58%, which could be viewed as excessive promiscuity. However, out of all of the ∼1 million enhancer gene connections in GeneHancer, a substantial subset (46.8%) are predicted without resorting to the nearest neighbor criterion. When focusing on the set of ∼94 000 elite enhancers, an even higher fraction of gene associations (56.8%) are predicted without the nearest neighbor criterion. Finally, by definition, none of the ∼40 000 double elite enhancer–gene connections is predicted by distance only. All of these observations highlight the fact that GeneHancer has a pronounced additional value beyond the most simple association of enhancers with their two closest genes. We finally note that adding proximity as an enhancer–gene association method does not appreciably increase promiscuity, as the average number of genes per enhancer only went up from 2.96 to 3.58. Still, in order to prevent an undesirable effect of the distance criterion on the gene–enhancer score, our distance-based scores are designed to have an attenuated contribution to the total gene–enhancer scores. The result is that the score distribution is only minimally affected by adding the distance criterion (Supplementary Figure S9).

## Conclusion

This article defines a path towards attaining knowledge in a realm that is strongly reliant on predictive algorithms. Obviously, extreme care should be exerted in utilizing and interpreting the ensuing data. A point of strength of our approach is source unification, which allows one to augment the confidence in predicting regulatory elements and their target genes. In particular, the capacity to define a high confidence ‘double elite’ subset encompassing ∼30k enhancers with ∼14k target genes represents a powerful genome interpretation tool. One could compare the utility of such a higher-confidence functional enhancer set to the popular gene prediction methods which have generated significant progress in disease gene discovery (exemplified by Refs 80–82 among ∼500 papers). Future improvements in GeneHancer will include the integration of new data sources, for both enhancer annotations (38, 39) and enhancer–gene associations, e.g. novel chromatin contact maps (83) and topologically associated domains (84). Such progress would help further overcome some of the inherent uncertainties and limitations, towards the goal of bringing integrated enhancer databases such as GeneHancer to a status closer to that of protein-coding gene compendia.

## Supplementary Material

Supplementary Data
