# Supplementary material for: GeneHancer: genome-wide integration of enhancers and target genes in GeneCards
Source: Database (Oxford). 2017 Apr 17;2017:bax028. doi: 10.1093/database/bax028 (PMC5467550; doi:10.1093/database/bax028)
Supplement: Supplementary Data [file bax028_Supp.zip › bax028-suppl_data/GeneHancer Supplementary File.docx]

## Supplementary methods

### eQTL analysis

eQTL data were obtained from GTEx (V6) ((1), <http://www.gtexportal.org>). The eQTL dataset used comprised cis-eQTLs within 1Mbp of the TSS of the associated gene. It included only eQTLs that passed GTEx significance criteria as described in <http://www.gtexportal.org/static/doc/analysis/Portal_Analysis_Methods_v6_08182016.pdf>. We combined the data from all tissues and kept the eQTL with the lowest p-value per genomic position. This was done in order for the eQTL data to be compatible with data from other sources. The GTEx data were transferred to BED format and converted to genome build hg38 using CrossMap. Only positions that mapped unambiguously were kept. BEDtools was used to map eQTLs onto regulatory elements. The eQTL-associated genes were symbolized in accordance with the GeneCards symbols based on the Ensembl gene identifier and the gene symbol as provided by GTEx, which was also done in the analyses described below.

Connections between genes and regulatory elements are made when one or more eQTL falling within the regulatory element are significantly associated with a candidate target gene. If several eQTLs fall within the same regulatory element and are associated with the same gene, the lowest p-value is used for the gene-regulatory element connection. This strategy eliminates potential problems resulting from high linkage disequilibrium between eQTLs.

Cognate analysis: we tested the eQTL dataset against a list of promoter-gene associations. Cognate rates were based on matches between gene symbols of genes associated with eQTLs and genes associated with a promoter (based on distance) i.e. when an eQTL falling in a promoter was linked with the gene with which the promoter was associated, it was counted as a match. The promoters utilized for the cognate analysis were taken from the Ensembl regulatory build (version 82). They were associated with protein-coding genes from the GeneCards database based on distance from the gene TSS. The midpoint genomic location of the promoter was calculated, and distance from the TSS measured. If a promoter midpoint was ≤2Kb from the TSS location it was defined to be linked to the gene. Only promoters that had unique gene connections were used.

To test for the possible enrichment of eQTLs in enhancers, we compared the occurrence of eQTLs in enhancers to that of non-eQTL SNPs; we used unique single nucleotide variant positions from dbSNP (human_9606_b147_GRCh38p2). Fisher’s exact test was performed to assess significance.

### Transcription factor co-expression analysis

To establish gene-enhancer connections, we performed cross-tissue expression correlation as described in (2), between candidate genes surrounding the enhancer (±1Mbp from TSS) and TFs whose binding sites are present within that enhancer. The *corr* function in Matlab was used to compute Pearson’s Correlation Coefficients and corresponding two-sided p-values for each TF-gene pair. This dataset was filtered by a threshold of r>0.75, using the 60 least abundant TFs (based on the number of enhancers containing their binding sites, Fig. S5). The rationale here is that highly ubiquitous TFs are less discerning for gene specificity, thus have the potential of enhancing noise. The p-values for each of the gene-enhancer associations resulting from different TFs were joined using Fisher's combined probability test (see Methods, “Gene-enhancer association and scoring”) to generate a single TFBS co-expression p-value. We excluded the trivial autocorrelation cases in which an enhancer contains a TFBS for a TF that also constitutes a potential target gene, as the correlation for every such case will by definition be equal to 1. This reflects an inherent incapacity of the TFBS method to identify true cases of auto-regulation. However, all other four association methods can discover such cases, and our records show 1,071 auto-regulating enhancers, involving 74% of all of the TFs analyzed.

To identify TFBSs within each enhancer, we mined ENCODE genome-wide ChIP-Seq peaks for 204 TFs and 85 biosample types (Table S6). We utilized only ENCODE inferences taken from file type “bed narrowPeak”, with output type “optimal idr thresholded peaks”, i.e. obtained via the irreproducible discovery rate (IDR) algorithm (3). Genome coordinates of the peaks were converted to hg38 with CrossMap and mapped onto the candidate enhancers with BEDtools. For each file, we used the top 75% of the peaks, based on the signalValue score, which is the sequencing depth-related score also used by the IDR procedure. Gene expression data were obtained from the GTEx project (V6) for 51 types of samples (tissues and cells), excluding transformed fibroblasts and EBV-transformed lymphocytes (1).

Validation tests for the TF co-expression method were performed by comparing the correlation distribution between known TF-target gene pairs and randomized control pairs. One validation set was 7,890 literature-curated TF-target gene pairs from TRRUST (4), which involve 742 TFs and 2,336 genes. For a second validation set we employed pairs of promoters and their target genes as described in the eQTL analysis validation paragraph in the Supplementary Methods. We then identified the TFBSs within each promoter as described for enhancers above. This allowed us to define a second set of 296,588 TF-gene pairs. For each TF-gene pair within the two validation sets we computed the tissue expression Pearson’s Correlation Coefficient as described above. Subsequently, the distribution of pairwise correlation coefficients was computed for each validation set. Finally, as randomized controls, we computed similar distributions for all possible TF-gene pairs in each set, excluding the known pairs. The Pearson’s correlation coefficients were Fisher-transformed for comparing the real and control distributions by the Wilcoxon rank sum test (using the *ranksum* function in Matlab).

### FANTOM eRNA data analysis

Data from the FANTOM5 atlas of active enhancers (5) were downloaded on 1 May 2016. FANTOM5 data were used in two ways: a) for identifying eRNA (enhancer RNA) -based enhancers (from the permissive_enhancers.bed dataset), and b) for establishing gene-enhancer connections based on a pairwise correlation between the eRNA expression and the TSS expression (from the enhancer_tss_associations.bed dataset).

The eRNA-TSS connections dataset contained associations between eRNAs and TSSs that were at most 500,000 bp apart from each other. Additionally, the dataset included only associations that survived a Benjamini-Hochberg FDR threshold of 10^-5^. The dataset included Pearson’s correlation coefficients (r values) for each eRNA-TSS pair. We used these to generate two-sided p-values using the Student’s t-distribution: each r value was converted into a t statistic using the following formula:
 $t=r\sqrt{\frac{n-2}{1-r^{2}}}$

and compared against a distribution of *n-2* degrees of freedom, where n was the number of cell type and tissue groups (so-called facets) used to generate the eRNA-TSS expression correlation coefficients (n=110). The p-values were generated in Matlab using the *tcdf* function. Each candidate enhancer containing FANTOM enhancers inherited the gene associations of its FANTOM components, taking the minimal p-value in cases where multiple FANTOM enhancer elements belonging to a candidate enhancer were linked to the same target gene.

### Capture Hi-C

CHi-C data were obtained from E-MTAB-2323 (6) at the EBI ArrayExpress repository, using the TS5_CD34_promoter-other_significant_interactions.txt and TS5_GM12878_promoter-other_significant_interactions.txt files containing the subset of authors-defined significant interactions. Genome coordinates were converted to hg38 with CrossMap. The original CHi-C data included pairs of fragments, where one member is a promoter and the other is a genomic fragment undergoing interaction with the promoter (using resolution of ≤10kb and only interactions reported in both tested cell types). As the original data contains pairs of segments where one member of each pair was already mapped with a known promoter, we mapped the non-promoter members onto the candidate enhancers (using BEDtools), thereby creating potential links between promoters (hence, genes) and enhancers. In the original CHi-C dataset, each interaction was assigned a score, which was $Log\frac{observed}{expected}$ read count. The maximum score was used for a given gene-enhancer connection.

### Nearest neighbor links

Based on the rationale that nearest-neighbor connections are of high likelihood, a distance-based method for gene-enhancer association was utilized. This entailed connecting to each enhancer the immediately neighboring gene, but not farther than 1Mb, on each side. Note that for all other methods we also invoked only gene enhancer connections occurring at distances ≤1Mb in the latest genome build.

## Supplementary figures


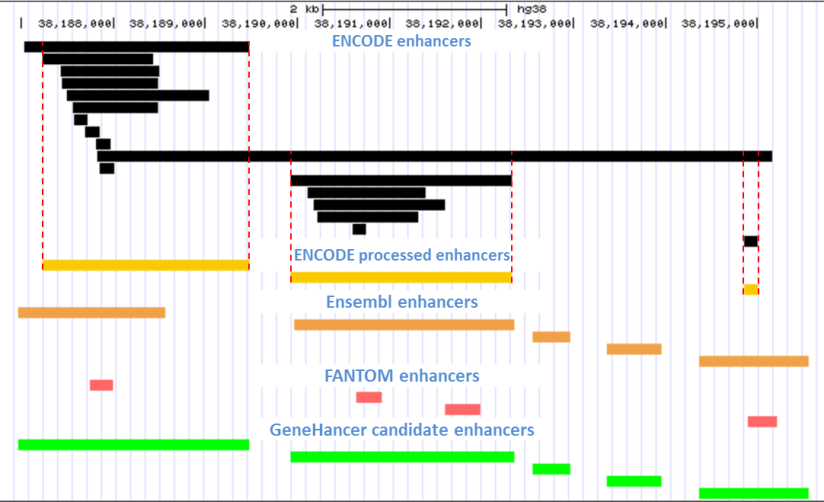


Fig. S1

ENCODE enhancer elements pre-processing. Example of a genomic region containing enhancer elements from ENCODE, Ensembl and FANTOM. First, ENCODE elements (black) that were reported separately for 46 cell lines and tissue types were pre-processed, resulting with a unique ENCODE enhancer list (yellow), comparable to other sources. This pre-processed dataset was further unified with other sources enhancer elements (orange, pink), to generate the GeneHancer candidate enhancers (green).


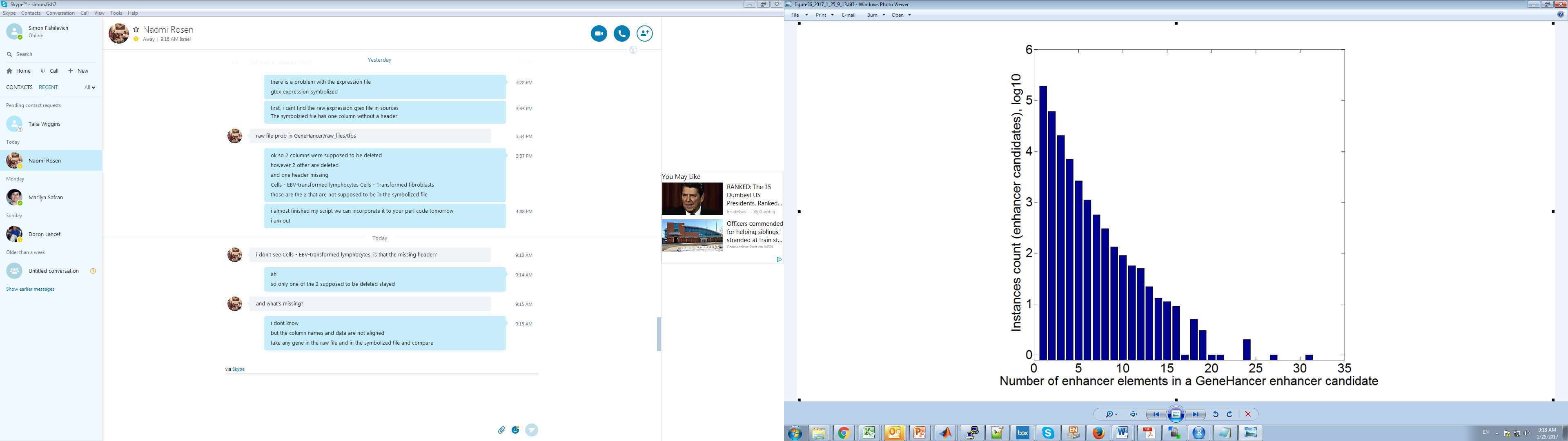


Fig. S2

Enhancer size. Distribution of the enhancer element counts (components) in each candidate enhancer.


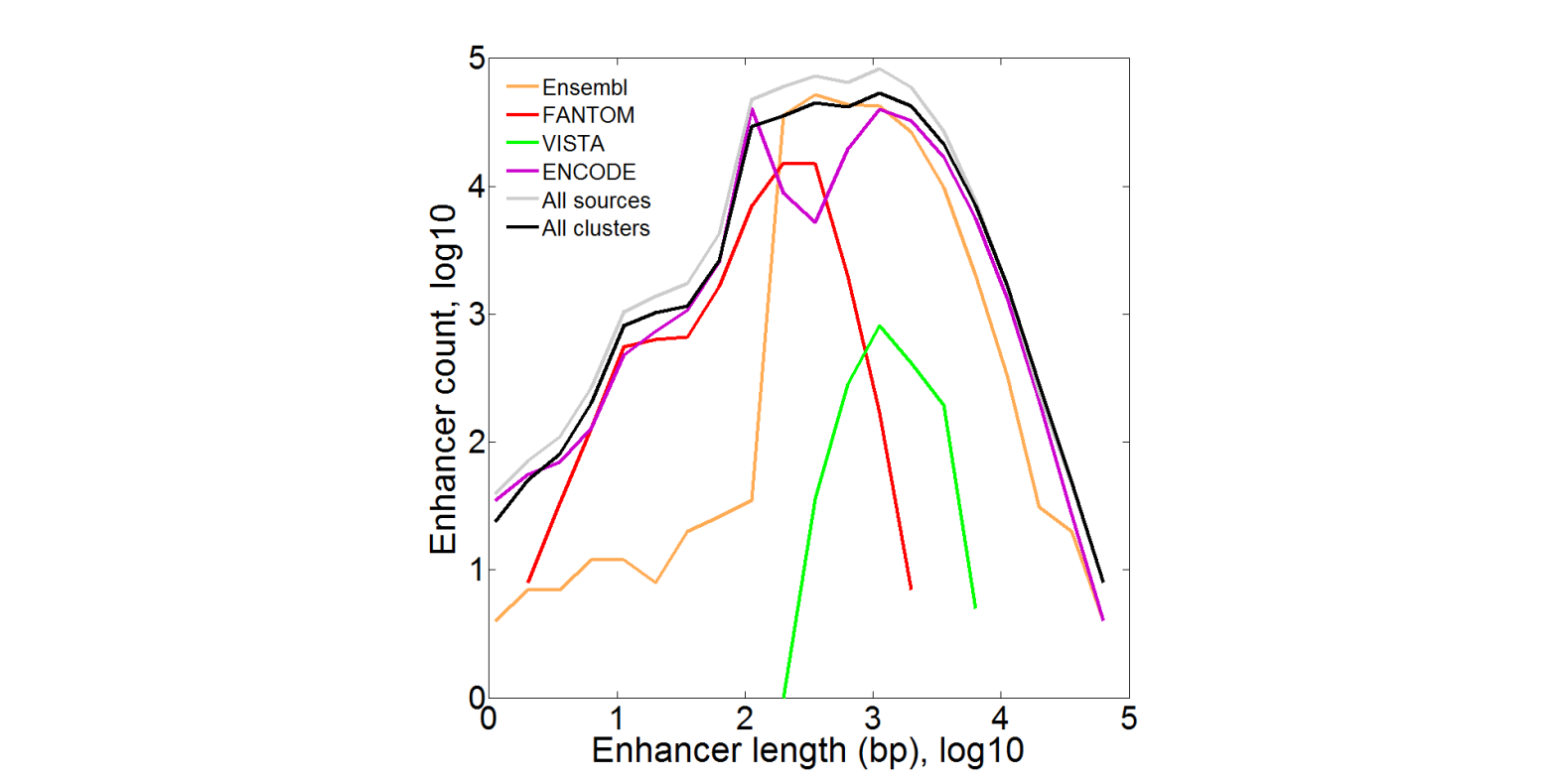


Fig. S3

Enhancer length. Distribution of the enhancer element and the candidate enhancer lengths.


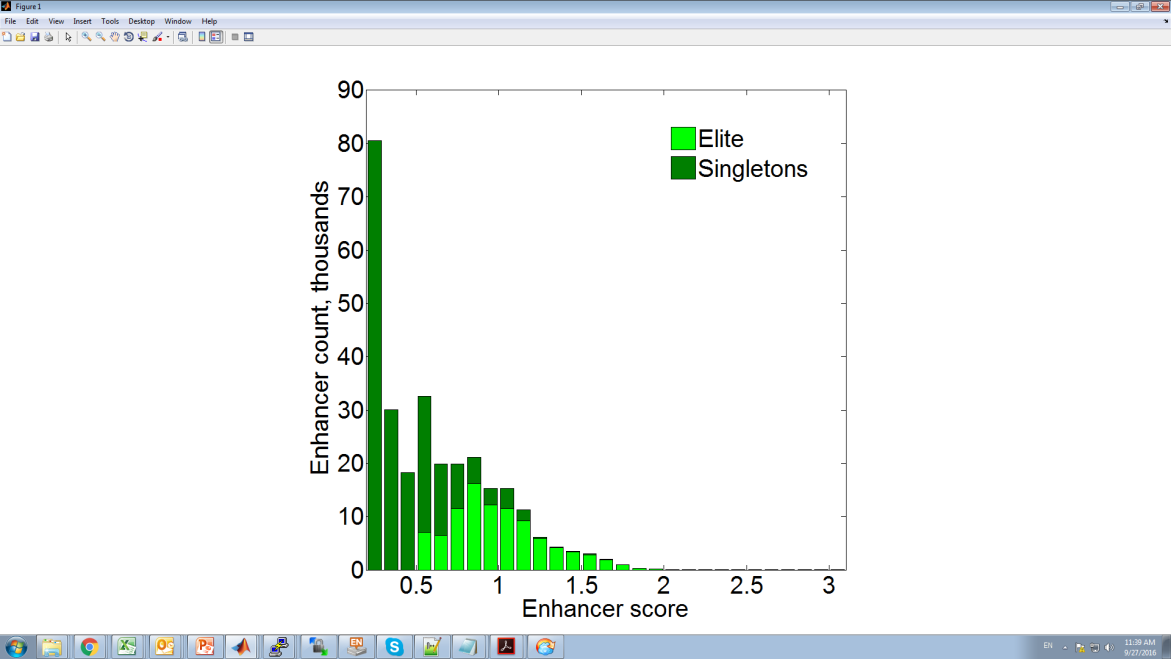


Fig. S4

Enhancer scores. Distribution of the candidate enhancers confidence scores.


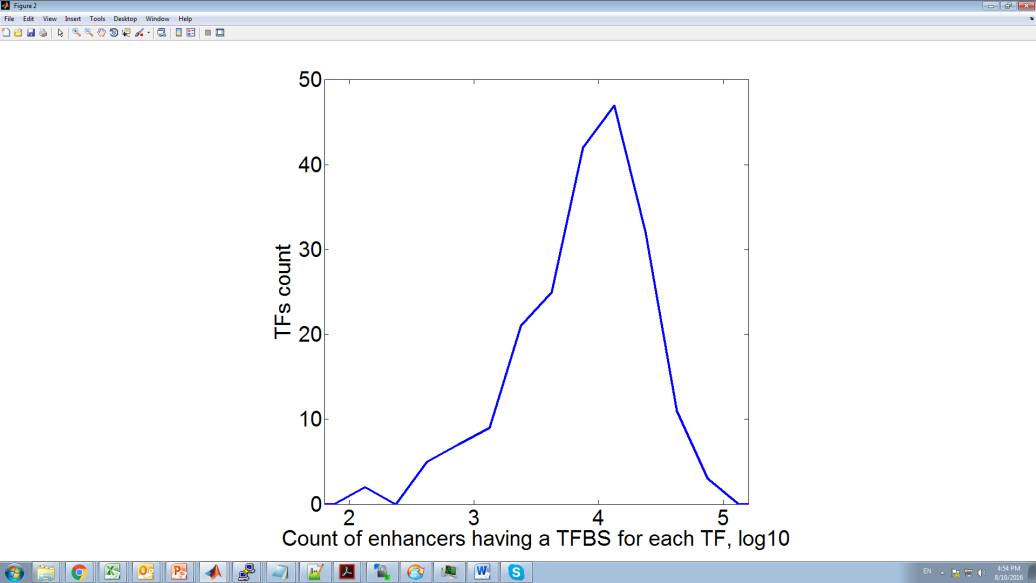


Fig. S5

Enhancers occupancy by TFBSs. Distribution of the TFBSs-based enhancer occupancy, defined as the number of enhancers having a TFBS for each of the 204 TFs (x-axis). Then, the count of TFs in every enhancer occupancy bin (y-axis) was used for the distribution.


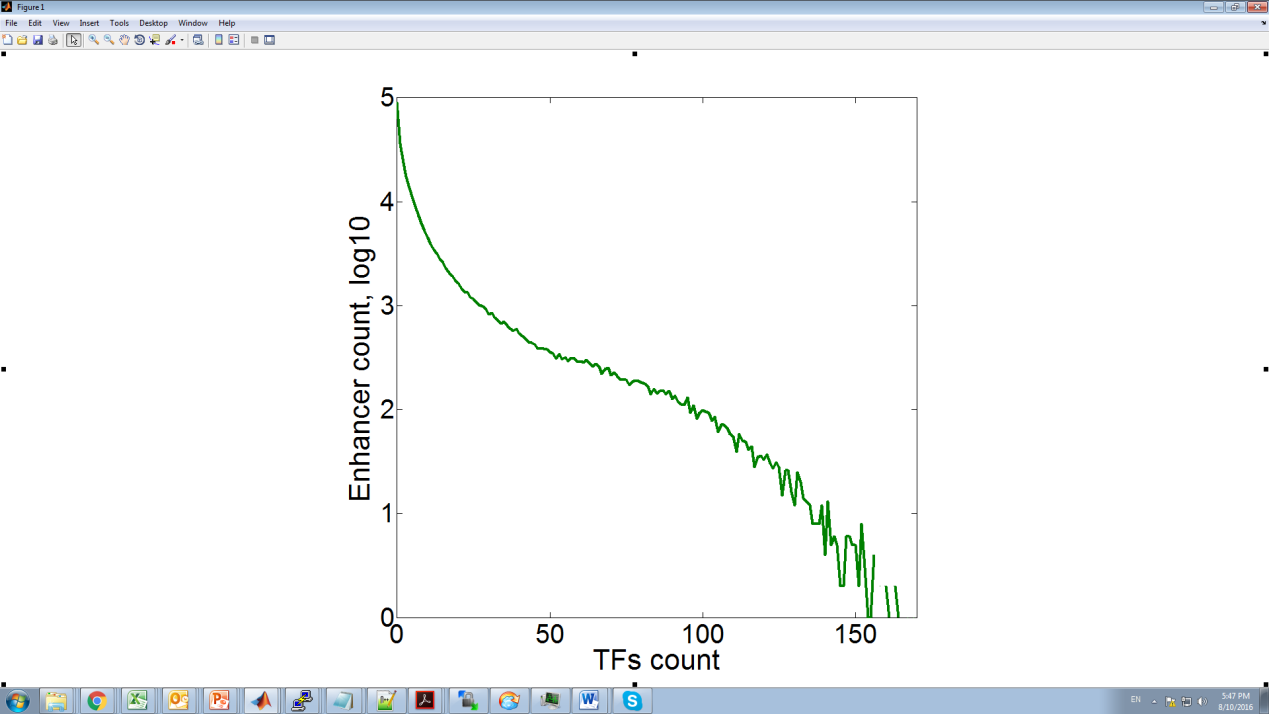


Fig. S6

TFs frequency in enhancers. Distribution of the number of TFs having a TFBS in each enhancer candidate. For every enhancer the number of TFs having a TFBS within the enhancer was calculated (x-axis). Then, the count of enhancers in every TFs number bin (y-axis) was used for the distribution.


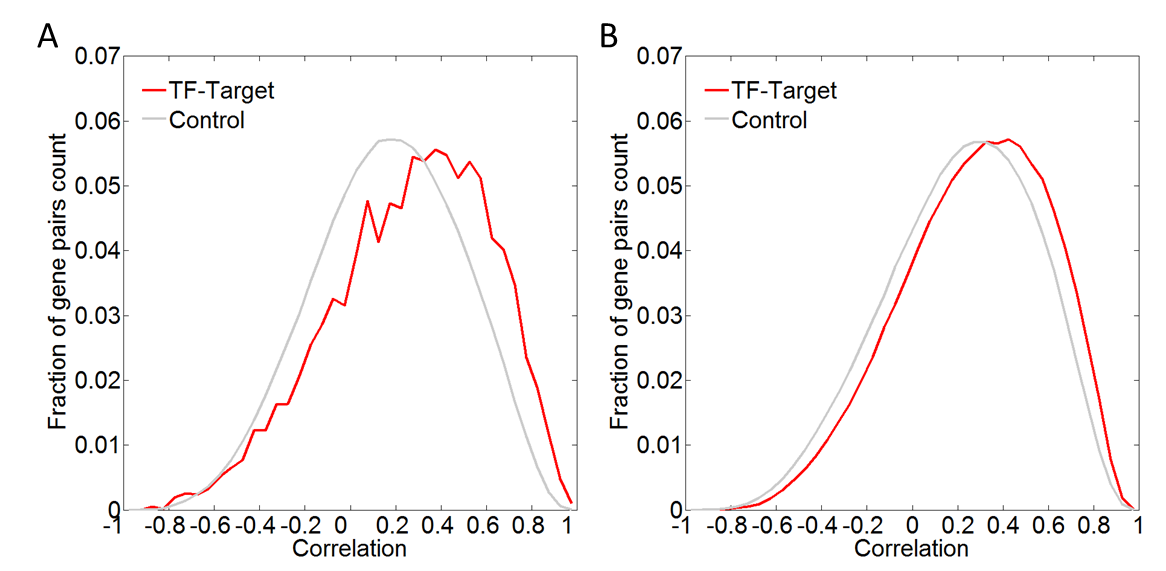


Fig. S7

Validation of the TF co-expression method. The distribution of the pairwise Pearson’s correlation coefficients is compared between an experimental validation set (red) and randomized controls (grey) (details in Supplementary Methods). (A) Known 7,890 TF-target gene pairs from TRRUST are compared with 1,987,965 control pairs. The experimental distribution has a higher median (0.30 vs 0.17 in the randomized control), with significance indicated by a Wilcoxon rank sum test, P<10^-5^. (B) Known 296,588 TF-gene pairs generated via gene-promoter relations are compared with 1,446,184 control pairs. The experimental distribution has a higher median (0.30 vs 0.24 in the randomized control), with significance indicated by a Wilcoxon rank sum test, P<10^-5^.


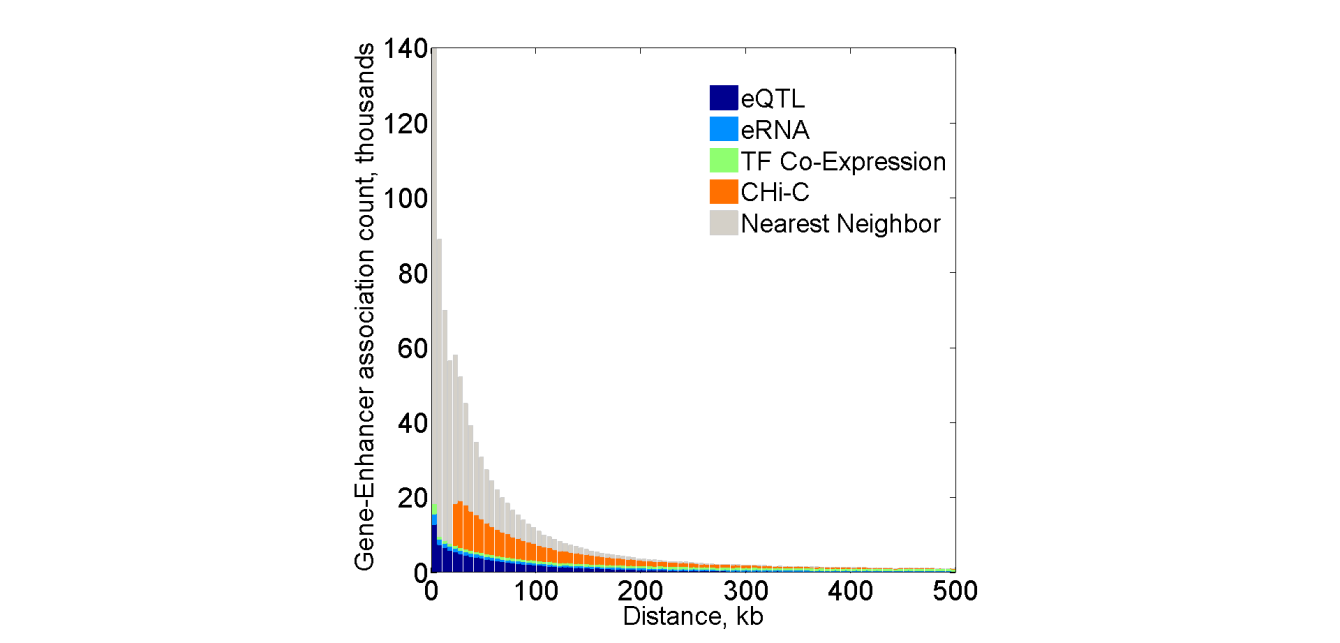


Fig. S8

Gene-enhancer association distance distribution. Gene-enhancer distance was calculated for each of the 1,102,033 gene-enhancer evidence associations and split by the evidence method type.


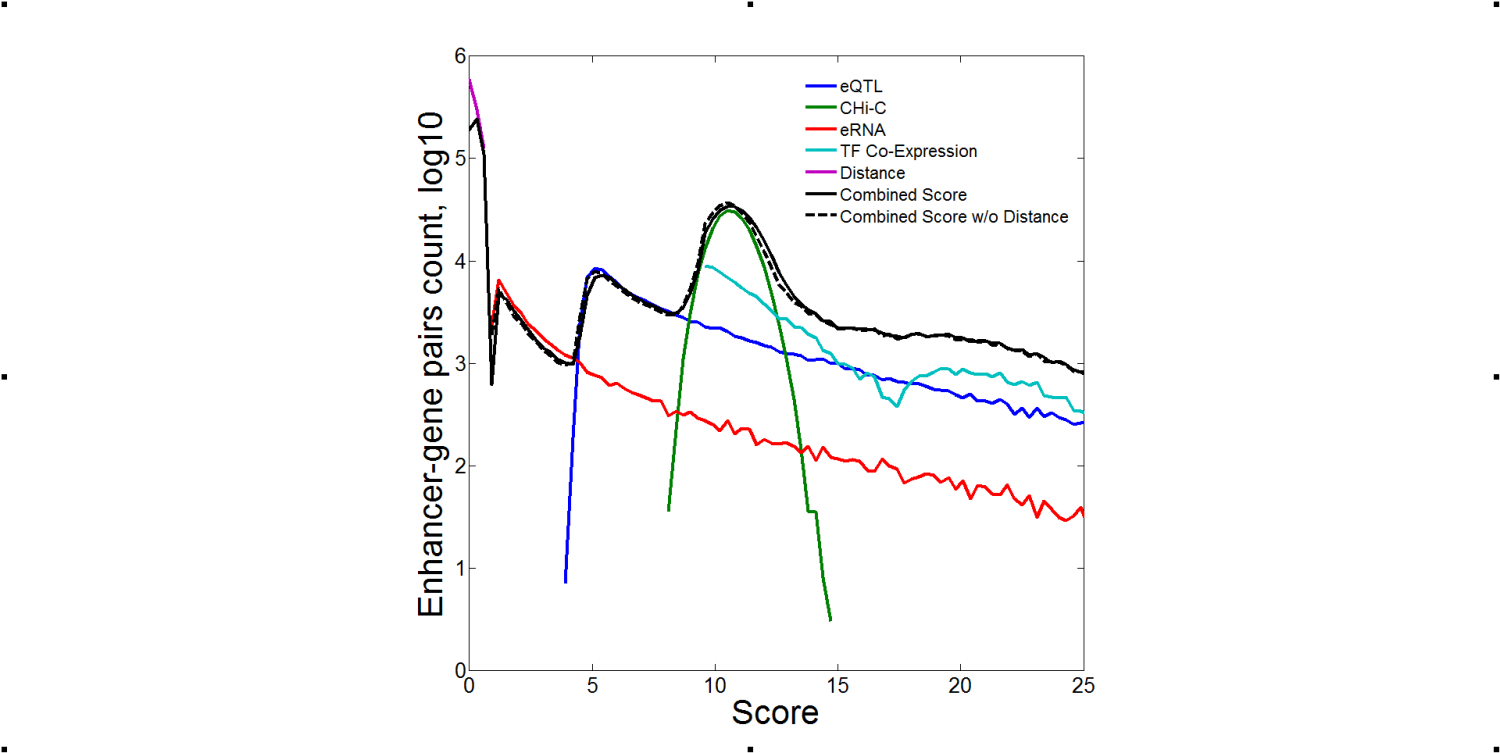


Fig. S9

Gene-enhancer scores. Distribution of the gene-enhancer association scores. X-axis units are shown as follows: –log10(p-value) for eQTLs, eRNA and TFs co-expression; log(observed/expected) for CHi-C; proportion for the distance (see Methods).


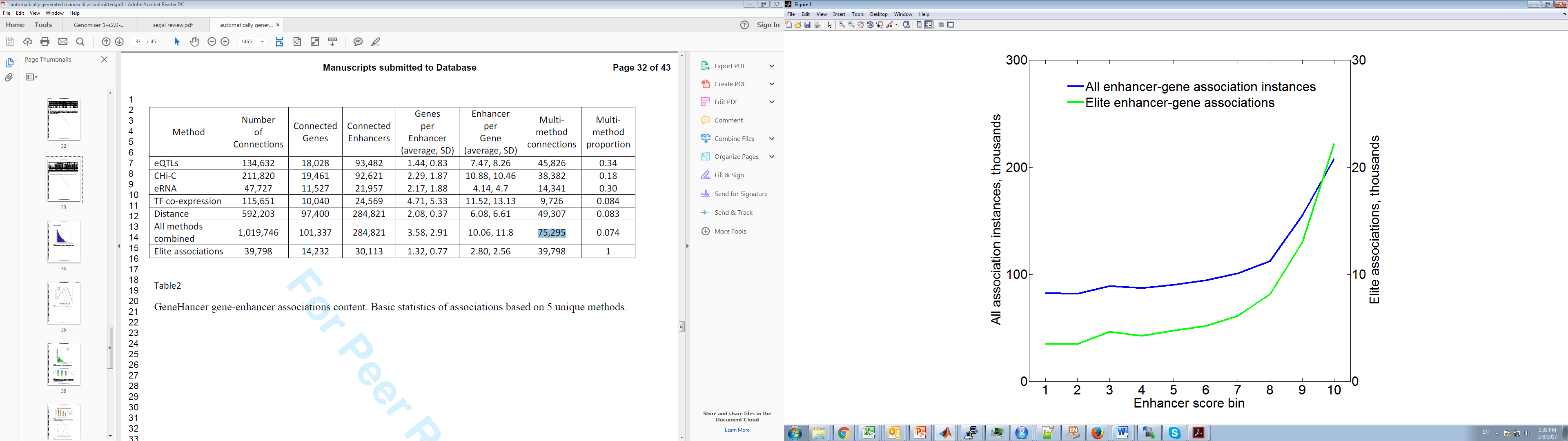


Fig. S10

Relationship between enhancer score and the enhancer-gene supporting evidence. All enhancers were binned into 10 equally populated bins. For each bin, we calculated (A) the total ammount of enhancer-gene associations (counting the association instances of all 5 methods) for the enhancers in the bin (blue); (B) the total ammount of elite enhancer-gene associations (supported by 2 or more methods) for the enhancers in the bin (green).


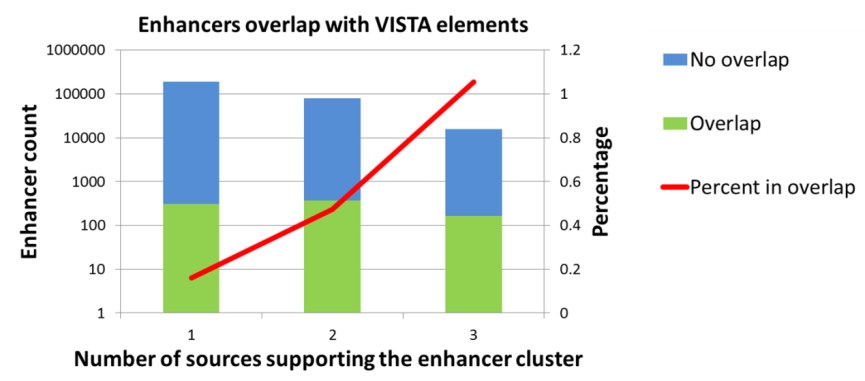


Fig. S11

Enhancer validation against the VISTA dataset. Enhancer candidates were split by the count of supporting sources (other than VISTA), and the overlap with the VISTA elements was calculated for each group.


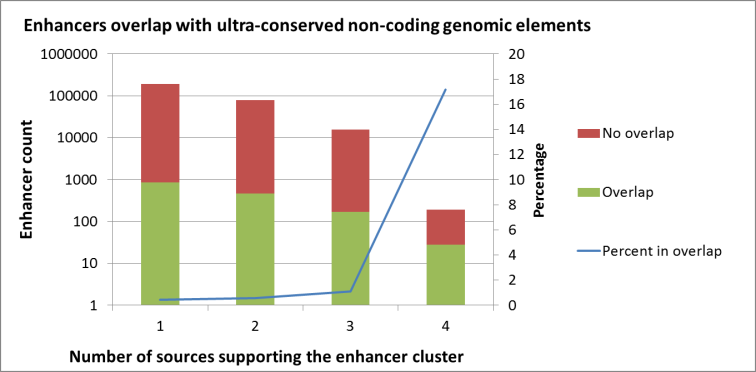


Fig. S12

Overlap with conserved regions. Enhancer candidates were split by the count of supporting sources, and the overlap with the ultra-conserved non-coding genomic elements was calculated for each group.


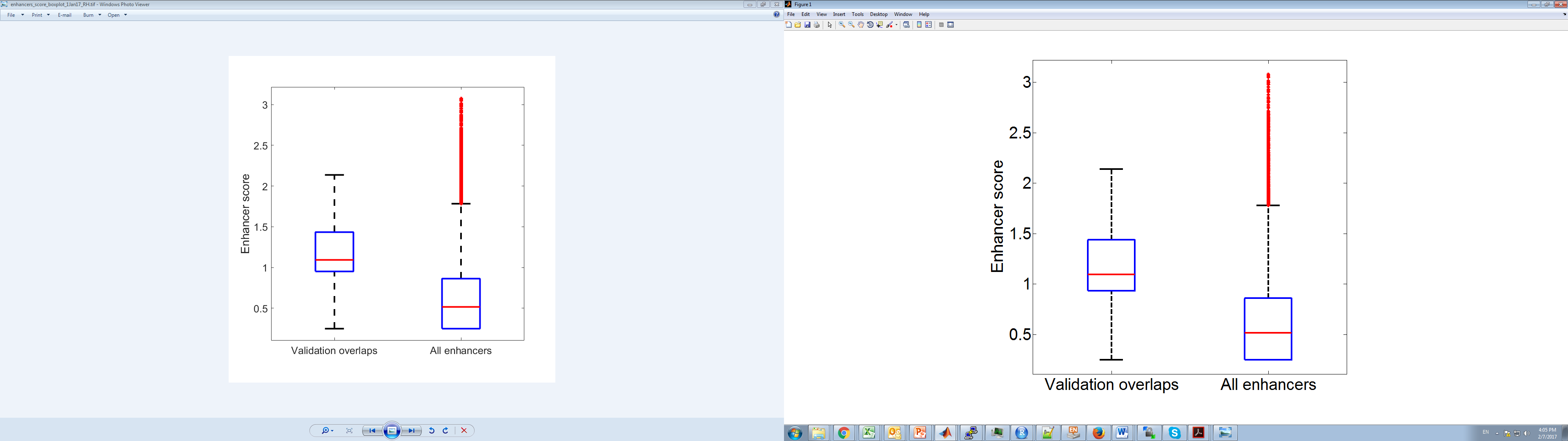


Fig. S13

Enhancer scores in the single enhancer studies comparison. Box plot of the enhancer scores of (A) 119 GeneHancer enhancers in overlap with the literature validation set; (B) All 284,834 GeneHancer enhancers. Interestingly, 55% of the literature overlap enhancers are elite, considerably more than the 33% elite fraction among all enhancers.


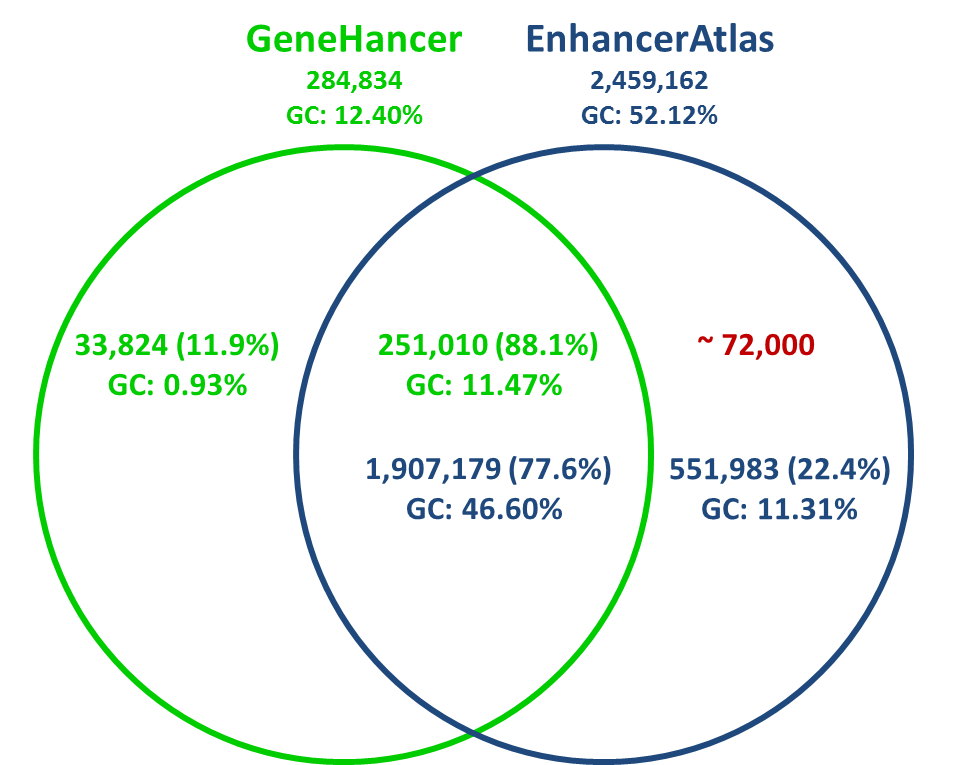


*Fig. S14*

Comparison of GeneHancer with EnhancerAtlas. Venn diagram of the overlaps between enhancer elements from GeneHancer and EnhancerAtlas. Green, counts and percentages for GeneHancer, Blue, the same for EnhancerAtlas. GC = Genome coverage.


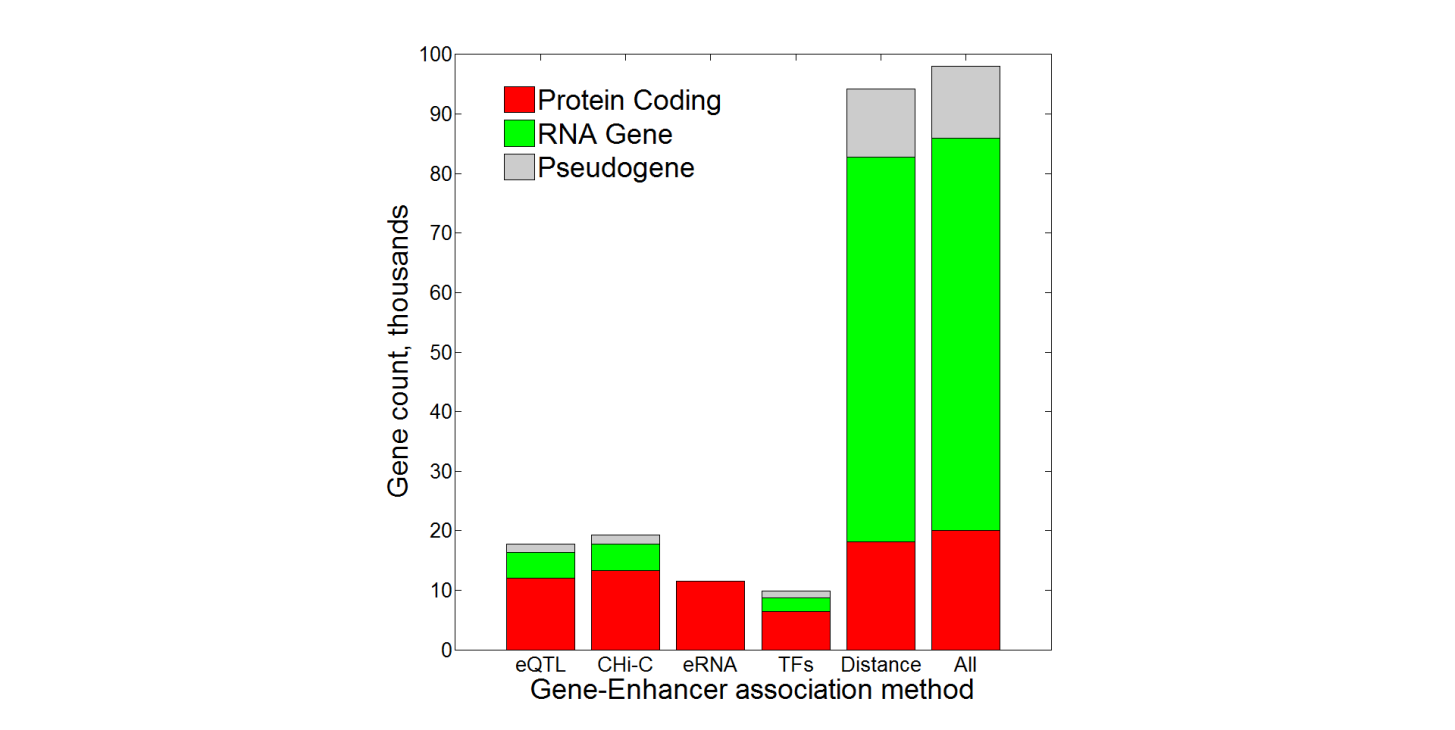


*Fig. S15*

Gene categories. Count of genes grouped by gene category for each of the gene-enhancer association methods.


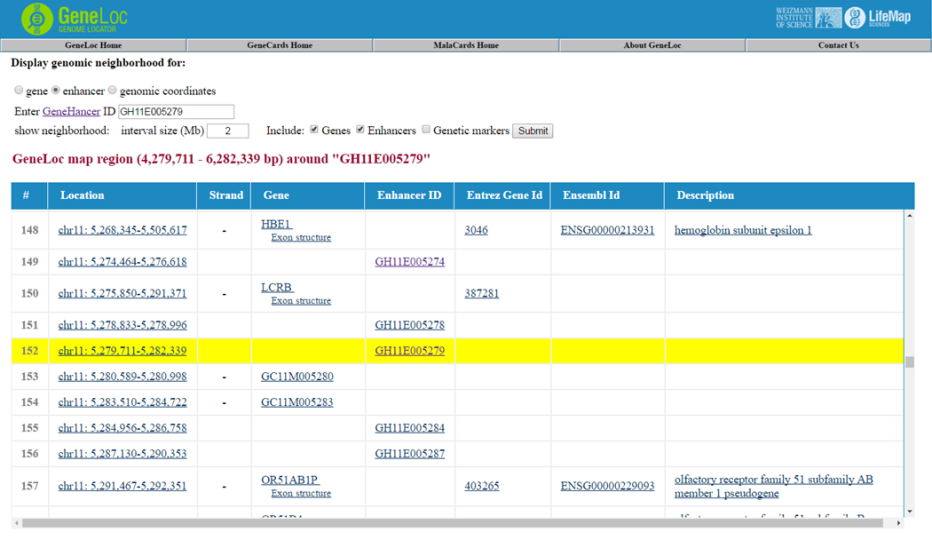


Fig S16

Screenshot of GeneLoc search results. This display exemplifies a genome map view centered on a specific enhancer (yellow highlight). Other modes of centering for search results are for a gene or for a chromosomal megabase coordinate. Rows represent genes or enhancers. Clicking on a GeneHancer ID shows genes associated with this enhancer in GeneCards. Clicking on a gene symbol leads to the relevant GeneCard. Clicking on the location coordinates of either gene or enhancer centers the tabulated map on the item. The screenshot was taken from GeneLoc version 4.3 website.

## Supplementary tables

|  | Enhancer Source | | | |
| --- | --- | --- | --- | --- |
|  | ENCODE | Ensembl | FANTOM | VISTA |
| Total candidate enhancers count | 166,565 | 191,097 | 35,421 | 1,715 |
| Multi source | 92,042 | 89,005 | 22,049 | 838 |
| Singletons | 74,523 | 102,092 | 13,372 | 877 |
| Multi source proportion | 0.55 | 0.47 | 0.62 | 0.49 |
| Overlap with VISTA (# of enhancers) | 685 | 654 | 195 | - |
| Overlap with VISTA (percentage out of the tested group) | 0.41 | 0.34 | 0.55 | - |
| Overlap with VISTA (percentage out of VISTA) | 0.40 | 0.38 | 0.11 | - |
| Overlap with VISTA (percentage out of VISTA normalized by the group genome coverage) | 0.0443 | 0.0531 | 0.2938 | - |
| Overlap with VISTA (Odds ratio) | 3.58 | 3.51 | 4.11 | - |

Table S1

Enhancer elements representation in GeneHancer. The number of candidate enhancers with evidence from each enhancer source is shown, including separated counts for candidates having support from a single source and from multiple sources. In addition, overlaps between each non-VISTA source enhancer elements and VISTA elements are presented.

|  | Enhancer source count  (non-VISTA) | | |
| --- | --- | --- | --- |
|  | Single | Double | Triple |
| Count of candidate enhancers | 190,292 | 78,204 | 15,461 |
| Overlaps with VISTA (# of enhancers) | 305 | 370 | 163 |
| Overlap with VISTA (percentage out of the tested group) | 0.16 | 0.47 | 1.05 |
| Overlap with VISTA (percentage out of VISTA) | 0.18 | 0.22 | 0.10 |

Table S2

Candidate enhancers overlap with VISTA elements. Excluding VISTA elements from the candidate enhancer set, the remaining candidate enhancers were split by the number of reporting non-VISTA sources of each enhancer. For each group of candidate enhancers (split by the count of reporting sources) the overlap with VISTA is presented.

|  | Source mutations | Used mutations | Regulatory elements | Enhancer overlaps | Gene target overlaps |
| --- | --- | --- | --- | --- | --- |
| Enhancer | 42 | 42 | 20 | 10 | 6 |
| Promoter | 142 | 142 | 58 | 39 | 34 |
| 5'UTR | 153 | 117^a^ | 54 | 39 | 36 |
| Total | 337 | 301 | 132 | 88 | 76 |

*Table S3*Validation using Mendelian regulatory mutations in Genomiser. Rows indicate subtypes of regulatory elements. ^a^ For 5’UTR not all mutations were used. This is because the data for 5’UTR include cases of transcription regulation as well as cases in which regulation is at the protein translation level. If the latter were included, our validation of target genes could generate false positives. This could happen when the genomic territory of an enhancer overlaps with the location of a translation control variation, and this specific 5’UTR belongs to our predicted target gene. Our elimination of 36 mutations that reside within our predicted enhancers for which the matching target genes are inferred based only on proximity significantly decreases the probability of such false positives.

We note that in this dataset the link between variant-containing regulatory element and gene is inferred via the relevant disease, as documented in OMIM. In rare cases the variant might lead to a disease via a different gene, not yet documented in OMIM. This would lead to (likely minor) inaccuracies in our target gene validation analysis.

## Supplementary tables provided as separate supplementary files

Table S4

Validation of GeneHancer predictions with 175 published cases of human functional regulatory regions confirmed by experiments (literature set). Each item in the table describes a case of a regulatory element and its gene target, as curated from the literature. Overlapping enhancers from GeneHancer are shown for each such record, along with respective gene targets.

Columns A-I describe the literature set, with genomic coordinates (hg38), element length, gene targets, PMIDs, group and type. ‘Group’ describes the literature set data source: ‘Genomiser’ - non-coding regulatory regions from (7); ‘Heart’ - in-vivo validated heart enhancers from the cardiac enhancer catalogue (8); ‘Literature sampling’ - our in-house curation effort. ‘Type’ describes the regulatory element type (enhancer/promoter/5’UTR). Columns J-N describe the GeneHancer candidate enhancers in overlap with the literature set elements, including genomic coordinates, length and gene targets. Columns O-S describe the comparison results, using 1/0 (true/false) status for each annotation, including: 1) Whether the literature element is in overlap with a GeneHancer enhancer; 2) Whether the gene target of the literature element is in the list of the GeneHancer enhancer target genes; 3) Whether the GeneHancer enhancer in overlap with the literature element is an elite enhancer; 4) Whether the gene-enhancer association in GeneHancer that matches the literature association is an elite association; 5) Whether the gene-enhancer association in GeneHancer that matches the literature association has a “double elite” status.

*Table S5*

A list of ENCODE Data Coordination Center (DCC) accession numbers of the predicted enhancer-like regions datasets mined for this study.

*Table S6*

A list of ENCODE Data Coordination Center (DCC) accession numbers of the ChIP-seq transcription factor binding sites datasets mined for this study.

## Supplementary acknowledgments

The Genotype-Tissue Expression (GTEx) Project was supported by the Common Fund of the Office of the Director of the National Institutes of Health. Additional funds were provided by the NCI, NHGRI, NHLBI, NIDA, NIMH, and NINDS. Donors were enrolled at Biospecimen Source Sites funded by NCI\SAIC-Frederick, Inc. (SAIC-F) subcontracts to the National Disease Research Interchange (10XS170), Roswell Park Cancer Institute (10XS171), and Science Care, Inc. (X10S172). The Laboratory, Data Analysis, and Coordinating Center (LDACC) was funded through a contract (HHSN268201000029C) to The Broad Institute, Inc. Biorepository operations were funded through an SAIC-F subcontract to Van Andel Institute (10ST1035). Additional data repository and project management were provided by SAIC-F (HHSN261200800001E). The Brain Bank was supported by a supplements to University of Miami grants DA006227 & DA033684 and to contract N01MH000028. Statistical Methods development grants were made to the University of Geneva (MH090941 & MH101814), the University of Chicago (MH090951, MH090937, MH101820, MH101825), the University of North Carolina - Chapel Hill (MH090936 & MH101819), Harvard University (MH090948), Stanford University (MH101782), Washington University St Louis (MH101810), and the University of Pennsylvania (MH101822). The data used for the analyses described in this manuscript were obtained from: the GTEx Portal on 29 May 2016.

## Supplementary references

1. Lonsdale, J., J. Thomas, M. Salvatore, et al., *The Genotype-Tissue Expression (GTEx) project.* Nature Genetics, 2013. **45**(6): p. 580-585.

2. Fishilevich, S., S. Zimmerman, A. Kohn, et al., *Genic insights from integrated human proteomics in GeneCards.* Database (Oxford), 2016. **2016**.

3. Li, Q., J.B. Brown, H. Huang, et al., *Measuring reproducibility of high-throughput experiments.* 2011: p. 1752-1779.

4. Han, H., H. Shim, D. Shin, et al., *TRRUST: a reference database of human transcriptional regulatory interactions.* Sci Rep, 2015. **5**: p. 11432.

5. Andersson, R., C. Gebhard, I. Miguel-Escalada, et al., *An atlas of active enhancers across human cell types and tissues.* Nature, 2014. **507**(7493): p. 455-61.

6. Mifsud, B., F. Tavares-Cadete, A.N. Young, et al., *Mapping long-range promoter contacts in human cells with high-resolution capture Hi-C.* Nat Genet, 2015. **47**(6): p. 598-606.

7. Smedley, D., M. Schubach, J.O. Jacobsen, et al., *A Whole-Genome Analysis Framework for Effective Identification of Pathogenic Regulatory Variants in Mendelian Disease.* Am J Hum Genet, 2016. **99**(3): p. 595-606.

8. Dickel, D.E., I. Barozzi, Y. Zhu, et al., *Genome-wide compendium and functional assessment of in vivo heart enhancers.* Nat Commun, 2016. **7**: p. 12923.
